# Multimodal tumor-homing chitosan oligosaccharide-coated biocompatible palladium nanoparticles for photo-based imaging and therapy

**DOI:** 10.1038/s41598-017-18966-8

**Published:** 2018-01-11

**Authors:** Subramaniyan Bharathiraja, Nhat Quang Bui, Panchanathan Manivasagan, Madhappan Santha Moorthy, Sudip Mondal, Hansu Seo, Nguyen Thanh Phuoc, Thi Tuong Vy Phan, Hyehyun Kim, Kang Dae Lee, Junghwan Oh

**Affiliations:** 1 Marine-Integrated Bionics Research Center, Pukyong National University, Busan, 48513 Republic of Korea; 2 Department of Biomedical Engineering and Center for Marine-Integrated Biotechnology (BK21 Plus), Pukyong National University, Busan, 48513 Republic of Korea; 3 Department of Otolaryngology – Head and Neck Surgery, Kosin University College of Medicine, Busan, Republic of Korea

## Abstract

Palladium, a near-infrared plasmonic material has been recognized for its use in photothermal therapy as an alternative to gold nanomaterials. However, its potential application has not been explored well in biomedical applications. In the present study, palladium nanoparticles were synthesized and the surface of the particles was successfully modified with chitosan oligosaccharide (COS), which improved the biocompatibility of the particles. More importantly, the particles were functionalized with RGD peptide, which improves particle accumulation in MDA-MB-231 breast cancer cells and results in enhanced photothermal therapeutic effects under an 808-nm laser. The RGD peptide-linked, COS-coated palladium nanoparticles (Pd@COS-RGD) have good biocompatibility, water dispersity, and colloidal and physiological stability. They destroy the tumor effectively under 808-nm laser illumination at 2 W cm^−2^ power density. Further, Pd@COS-RGD gives good amplitude of photoacoustic signals, which facilitates the imaging of tumor tissues using a non-invasive photoacoustic tomography system. Finally, the fabricated Pd@COS-RGD acts as an ideal nanotheranostic agent for enhanced imaging and therapy of tumors using a non-invasive near-infrared laser.

## Introduction

Theranostics, a single platform that provides an opportunity for therapy and diagnosis, is highly recognized to fight against various diseases^1,2^. Nanomaterials that absorb light in the near-infrared (NIR) region have gained considerable attention because of their promising application in both photothermal therapy (PTT) and imaging applications. The use of metal nanoparticles as a NIR thermal transducer has been well explored and where silver, iron, gold, platinum, copper, and upconversion nanoparticles are largely dominating^3^. The most commonly used gold nanomaterials diminished their plasmonic properties after long periods of NIR laser irradiation owing to their melting points. Recently, palladium, the low cost metal has been recognized in the biomedical field as a noble metal with remarkable stability and good catalytic and mechanical properties^4^. Palladium nanostructures are widely used in different applications as a catalyst for C-C bond formation and oxidation processes in the pharmaceutical field^5^. Moreover, palladium is used in clinical settings for prostate cancer and choroidal melanoma brachytherapy^6^. Only a very few of reports are available on the use of palladium nanostructures as prodrug activators, photothermal agents, and anticancer or antimicrobial agents^7^. However, the multifactorial interaction of metal nanoparticles with biological system causes toxic effects that impede the wide application of these nanostructures in the pharmaceutical field. Indeed, advances in the synthesis of nanoparticles and their surface engineering with amino acids, polymers, and other natural products have endowed them with biocompatibility, stability, and dispersity in physiological solution, as well as facilitating further modification for targeting ligand-based therapies^8^. Also, continued efforts have been made to develop surface modifications for safe therapeutic applications and clinical translations of metal nanoparticles.

Palladium nanoparticles (Pd NPs) with NIR absorbance have gained interest in PTT, a newly developed strategy that employs NIR laser photo-absorbers to generate heat under NIR laser irradiation^9^. PTT has many advantages over conventional chemotherapies, including high specificity, minimal invasiveness, precise spatial-temporal selectivity and effective eradication of tumor tissue^10–12^. Further, PTT can be combined with multimodal imaging to monitor or realize their therapeutic outcome. Recently, interest in photoacoustic tomography (PAT) has increased in biomedical fields due to its use in non-invasive imaging without ionizing radiation and tissue damage. PAT offers deep tissue imaging with high spatial and temporal resolution^13^. Nanoparticle-based contrast agents can produce an effective signal at the site of interest in PAT systems^14^. A continuous effort has been devoted to the development of PAT agents, particularly with gold nanoparticles due to their optical properties^15^. The PAT efficiency of Pd NPs has not been studied much, except the study by Chen *at al*.^16^. The NIR absorbance spectrum of Pd NPs allows their use in PAT applications for image-combined therapeutic application. In the NIR spectral region, the optical attenuation of tissue is usually low, which offers deep light penetration and precise imaging of the external contrast agent injected tissue.

Untargeted theranostic agents reduce therapeutic efficiency by non-specific accumulation in other tissues. There are two possible mechanisms to improve the tumor selectivity of nanoparticles: (i) passive targeting through enhanced permeability retention, and (ii) active targeting based on a specific cell-surface receptor with a ligand on the nanoparticles^17^. Integrins, a heterodimeric cell adhesion proteins involved in many mechanisms, including cell attachment, angiogenesis, and the metastasis of solid tumors, have been identified for active targeting of tumor tissue in clinical trails^18^. Among the different types of integrin, alphaV beta3 (α_v_β_3_) integrins are recognized as promising therapeutic targets because they are overexpressed during proliferation of tumor cells^19^. In the present work, we functionalized Pd NPs with an RGD (arginine-glycine-aspartic acid) motif that can bind to α_v_β_3_ integrins on the cell surface and possibly increase the homing of the phototherapeutic agent to the tumor site.

Chitosan is a natural, biodegradable, nontoxic, cationic carbohydrate polymer widely used in pharmaceutics, cosmetics, and the food industry^20^. Chitosan is largely obtained from chitin of crustacean’s shell and it is composed of β-1,4-linked d-glucosamine. In its native form, chitosan has low solubility in acid-free aqueous medium, which has restricted its application in the pharmaceutical field. In recent years, intensive studies have led to the development of a low-molecular-weight and water-soluble chitosan oligosaccharide (COS) from chitosan. Owing to its unique properties, COS has become as an excellent candidate for various biomedical applications, including drug delivery, gene delivery, and tissue engineering^21^. In the present work, the surface of Pd NPs was modified with a COS polymer (Pd@COS NPs), which confers biocompatibility and enables further functionalization with other molecules of interest via conventional coupling chemistry using the amine and hydroxyl groups present in the polymer.

Breast cancer, one of the most common cancers among women, leads to death worldwide due to therapeutic resistance to the traditional chemotherapy^22^. Triple-negative breast cancer (TNBC) is a subtype of breast cancer that is characterized by negative expression of progesterone, estrogen, and epidermal growth factor receptor 2 and it can be metastasis aggressively^23^. TNBC represents 10–15% of breast cancers and patients with this cancer subtype have poor outcomes with clinical chemotherapy. The TNBC cell line MDA-MB-231, which has been characterized for integrin α_v_β_3_ positive expression^24^, which was chosen as a model cell line to study the targeting ability of RGD peptide conjugated NPs in the present study.

In the present work we synthesized COS-coated biocompatible Pd NPs and functionalized them with RGD peptide (Pd@COS-RGD) for effective accumulation in breast cancer cells. Further, the NIR-based photothermal ablation and PAT imaging efficiency of the formulated particles were examined using *in vitro* and *in vivo* models.

## Results and Discussion

### Nanoparticles system

**Figure 1:**
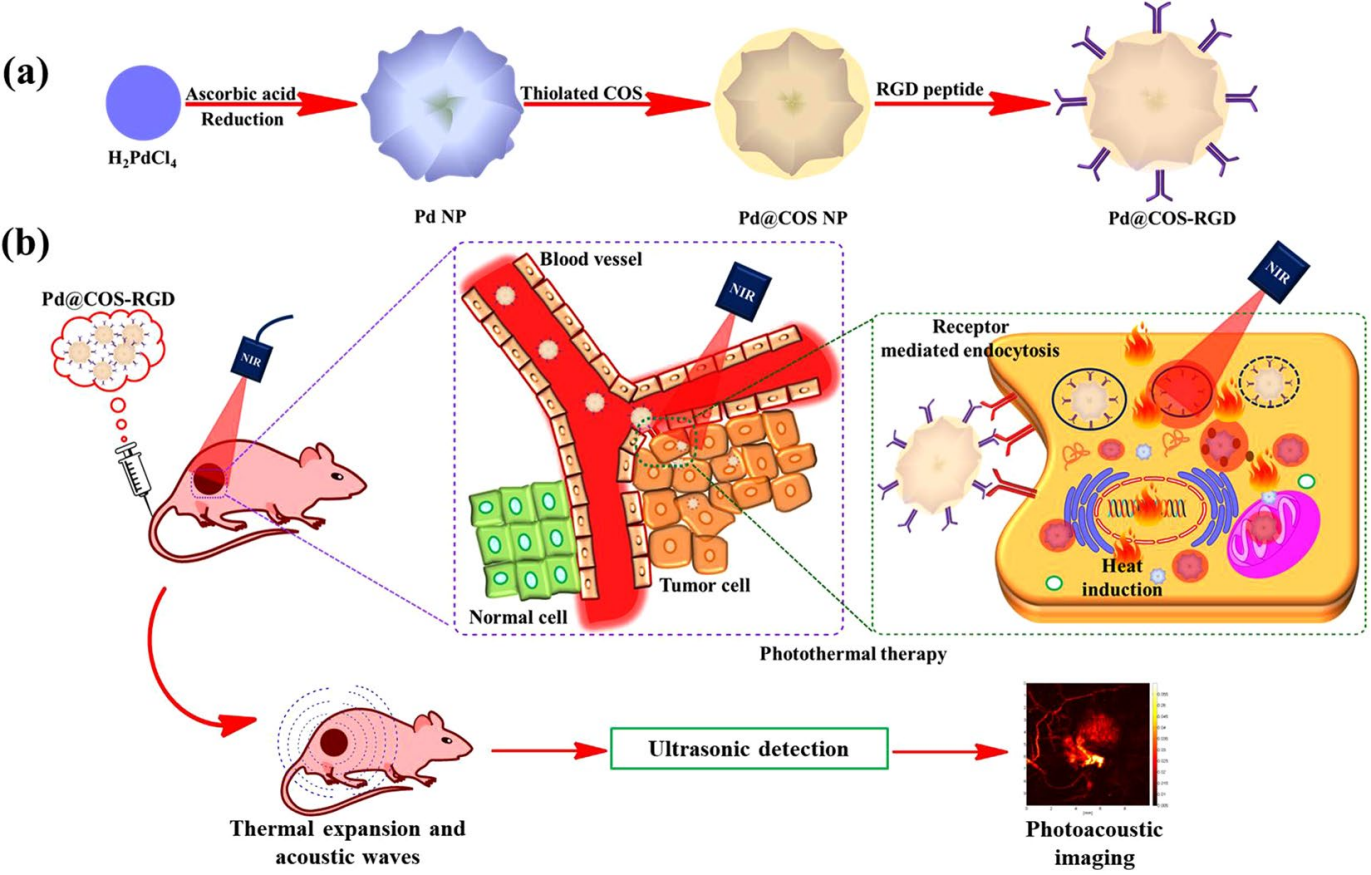
(**a**) A scheme showing the preparation of Pd NPs and further surface coating with thiloated chitosan oligosaccharide (Pd@COS NPs) and finally functionalization using RGD peptide (Pd@COS-RGD). **(b)** A systematic illustration showing the photothermal ablation and photoacoustic imaging of tumor tissue using Pd@COS-RGD.

The palladium nanostructures have been recognized in biomedical fields for their remarkable optical and catalytic properties. The facile preparation of Pd NPs and step-wise surface modification with COS and RGD peptide are shown in Fig. 1a and Fig. S1. First, thiolated COS was coated onto the surface of Pd NPs via ligand exchange approaches to obtain Pd@COS NPs. Second, maleic anhydride was conjugated onto the surface of Pd@COS NPs via the ring opening reaction. Maleic anhydride groups can react with the hydroxyl (-OH) and secondary amino (-NH) groups present in COS polymeric units to produce “ene” groups (Pd@COS-COOH NPs). Finally, cyclic RGD peptide units were successfully conjugated onto Pd@COS-COOH NPs using “thiol-ene click chemistry” by the reaction between the thiol groups of the RGD peptide and the “ene” part of Pd@COS-COOH NPs to obtain Pd@COS-RGD, which is the final product (Fig. S1). The receptor-mediated accumulation of Pd@COS-RGD in tumor cells and their dual-mode application for PTT and PAT imaging are shown in Fig. 1b.

### Characterization of nanoparticles

**Figure 2:**
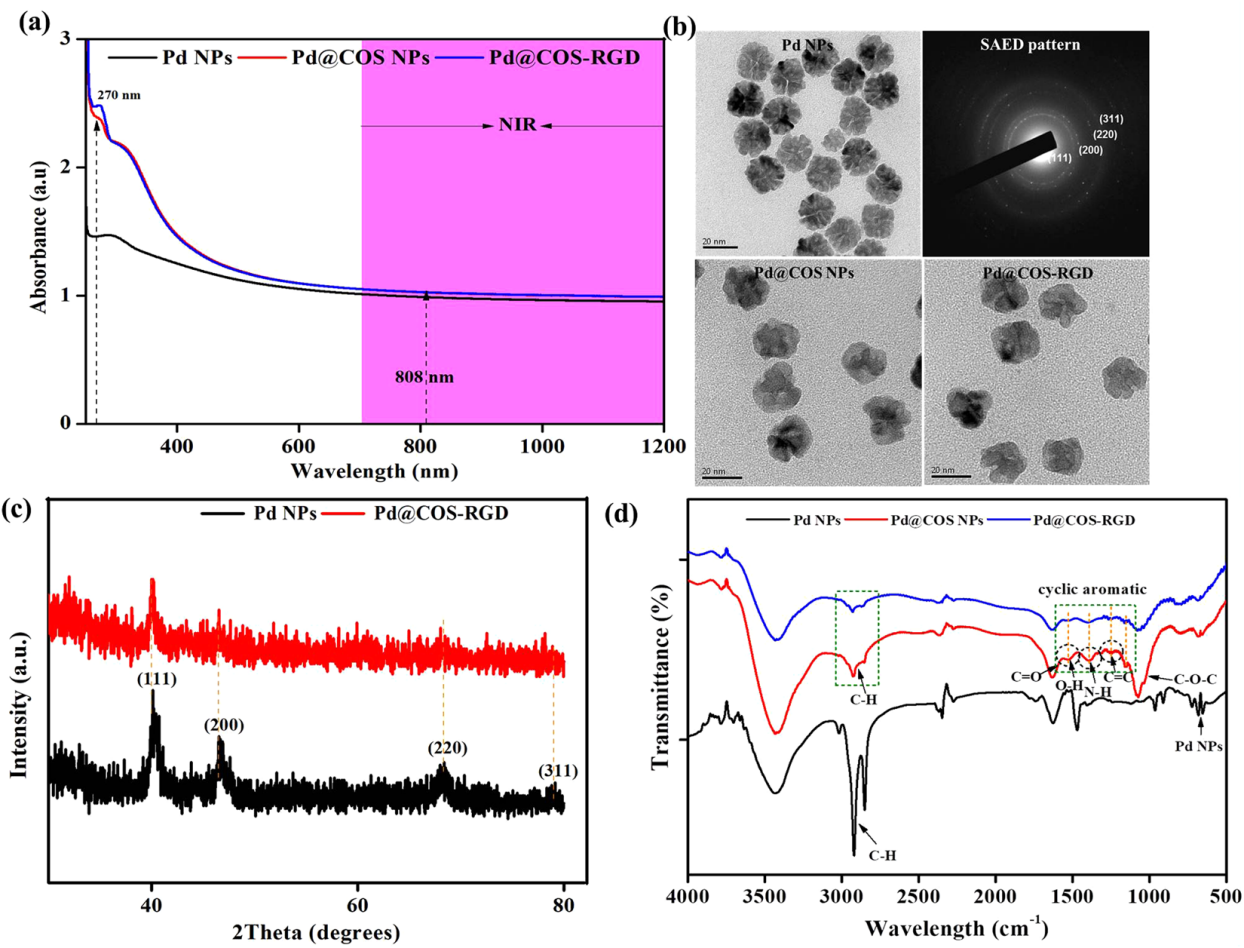
(**a**) UV-Vis absorbance spectrum of Pd NPs, Pd@COS NPs, and Pd@COS-RGD dispersed in water. **(b)** TEM images of Pd NPs with different modifications along with SAED patterns. **(c)** The X-ray diffraction (XRD) patterns in the 2θ range 20–80° of initial Pd NPs and final Pd@COS-RGD. **(d)** FTIR spectrum of freeze-dried Pd NPs, Pd@COS NPs, and Pd@COS-RGD.

In the present study, porous Pd NPs were synthesized by following a seed-mediated growth protocol in aqueous solution with cetyltrimethylammonium chloride (CTAC) as the stabilizing agent, as described by Wang *et al*.^25^. The absorbance spectrum of Pd NPs observed from the UV-Vis to the NIR region (Fig. 2a), which qualifies the Pd NPs as potential photothermal agent using a NIR laser. Figure 2b reveals that the size of the Pd NPs was relatively uniform, with a flower-like spherical shape containing porous structures. The size distribution of the as-synthesized Pd NPs fell between 18 and 26 nm, with an average size of 22.26 ± 0.97 nm (Fig. S2a). Selected area electron diffraction (SAED) analysis shows the crystalline nature of the synthesized particles (Fig. 2b). The lattice fringes detected in the SAED pattern [(111), (200), (220), and (311)] reveals the crystal planes of Pd NPs. In addition, the XRD pattern of the synthesized Pd NPs shows peaks at 40.1°, 46.5°, and 68.3°, which correspond to the (111), (200), and (311) crystal planes (Fig. 2c), supporting the crystalline nature of Pd NPs (JCPDS No. 46–1043). The peak intensity at 40.1° (111) was most intense, compared with the other reflection peaks, which may reflect the preferred direction of nanocrystal growth^26^.

### Surface modification of Pd NPs with COS

COS-SH was prepared by the formation of amide bonds between the NH_2_ groups of COS and the carboxyl group of thioglycolic acid (TGA) using 1-ethyl-3-(3-dimethylaminopropyl) carbodiimide (EDC) as a catalyst. The self-assembly of thiol group of polymer on metal surfaces is well known process^27^. The COS-SH was soluble in water and the thiol group content was found to be 63% using Ellman’s protocol. The Pd NPs surface was modified with COS-SH to reduce their cytotoxic effect and allow further functionalization with the RGD peptide. CTAC molecules were replaced with COS-SH, which shifted the absorbance intensity of Pd NPs slightly higher. The peak absorbance at 270 nm could be attributed to COS (Fig. 2a). The RGD peptide was linked to the free hydroxyl and amino groups of the COS present on the Pd@COS NPs. The free amino group content of the Pd@COS NPs was calculated to be 276 ± 15 µmol/g using the 2,4,6-trinitrobenzenesulfonic acid (TNBS) method^28^. After conjugation of the RGD peptide, the number of free amino groups was reduced to 198 ± 17 µmol/g. The conjugation of RGD peptide was quantified using the BCA assay and that was predicted to be 13.98 RGD peptides on each Pd@COS NPs^29^. There was no difference in absorbance spectrum between Pd@COS NPs and Pd@COS-RGD after conjugation of the RGD peptide with Pd@COS NPs (Fig. 2a). As illustrated in Fig. 2b, the fringes of the Pd NPs were covered with COS and no variable changes were observed in the shape of the particle after conjugation with RGD peptide. The XRD pattern revealed that the crystalline nature of the particles was decreased after surface modification in Pd@COS-RGD (Fig. 2c). The reflection intensities of Pd@COS-RGD were reduced due to the functional moieties of COS present on the Pd NPs. This was also confirmed by keen observation of the TEM images of Pd@COS-RGD (Fig. 2b). The surface modification process does not affect the size distribution of Pd NPs significantly (Fig. S2a and b). The average size of Pd@COS NPs and Pd@COS-RGD was detected as 23.54 ± 1.25 nm (Fig. S2b) and 24.28 ± 1.29 nm (Fig. S2c) respectively. In addition, the zeta potential analysis showed changes in surface modification for each step (Fig. S2d). Initially, Pd NPs had a positive zeta potential of 13.1. Then, the positive value was increased to 17.52 in Pd@COS NPs due to the positive nature of COS^30^. After functionalizing Pd@COS NPs with RGD peptide, the final charge of the particles became14.65 (Fig. S2d).

Pd@COS-RGD NPs displayed excellent long-term stability in aqueous media for at least 6 months. No significant change in surface plasmon band position was observed during long-term storage in water (Fig. S3a). It showed that the Pd@COS-RGD was stable and no aggregation was found in aqueous solution. The aggregation of particles give rise to coupled plasmon bands at longer wavelengths^31^; however, these bands were not observed (Fig. S3a). The particle size remained same and no flocculation was observed during long-term storage in distilled water (Fig. S3b). The Pd@COS-RGD had good colloidal stability in different physiological solutions (Fig. S3c), which is consistent with reports that COS modifications has colloidal stability^32^.

Figure 2d presents the FTIR spectral analysis of the as-synthesized Pd NPs, the COS-coated Pd@COS NPs, and the RGD peptide-conjugated Pd@COS-RGD nanoparticles. As shown in Fig. 2d, the characteristic peaks at 2857 cm^−1^ and 2922 cm^−1^ indicate the presence of the–CH_2_ groups of CTAC, which was on the surface of the pristine Pd NPs. The stretching vibration peak present at 657 cm^−1^ implies the presence of the Pd NPs. The IR spectrum of Pd@COS NPs shows the disappearance of characteristic CTAC bands and the appearance of new peaks that provide evidenced that the Pd NPs were coated with COS via a ligand exchange reaction. The bands at 2857 cm^−1^ and 2921 cm^−1^ are due to the C-H stretching of the COS units. Specifically, the stretching vibration peaks at 1395 cm^−1^, 1527 cm^−1^, and 1075 cm^−1^ seen in the COS that was reacted with maleic anhydride units and then modified with TGA indicate the N-H, -O-H, and C-O-C groups present in the COS polymeric units on the Pd@COS NPS. In addition, the overlapping peaks that appeared at 1249 cm^−1^ and 1603 cm^−1^ indicate the presence of C=C and C=O groups in the Pd@COS NPs (Fig. 2d). Furthermore, the spectrum of Pd@COS-RGD showed that almost all of the prominent peak intensities become relatively decreased, which indicating that the thiolated RGD peptide units interacted with the Pd@COS NPs surfaces through the covalent bond formed by thiol-ene click reaction and hydrogen bonding (Fig. 2d).

Thermogravimetric analysis was performed to screen the coating of COS polymeric units and the further conjugation of RGD peptide units onto Pd NPs. As shown in Fig. S3d, all the samples show an initial weight loss of approximately ~6.2 wt.% at the temperatures of 100–170 °C due to physisorbed solvent or moisture. Furthermore, the second weight loss of about ~16.5 wt.% for the Pd NPs indicates the decomposition of CTAC present on the Pd NPs (Fig. S3d). In contrast, the COS-coated Pd@COS NPs and further RGD peptide-conjugated Pd@COS-RGD NPs shows a collective weight loss of approximately ~38 wt.% in the temperature range between 180 and 460 °C, indicating that the surface of the Pd NPs was coated with COS polymer (Fig. S3d). The further ~ 8 wt.% loss of Pd@COS-RGD indicated the decomposition of the RGD peptide conjugated COS polymer (Fig. S3d). The thermogravimetric data showed that a considerable amount of COS and RGD peptide units were incorporated in Pd@COS-RGD.

### Photothermal heating effect

**Figure 3:**
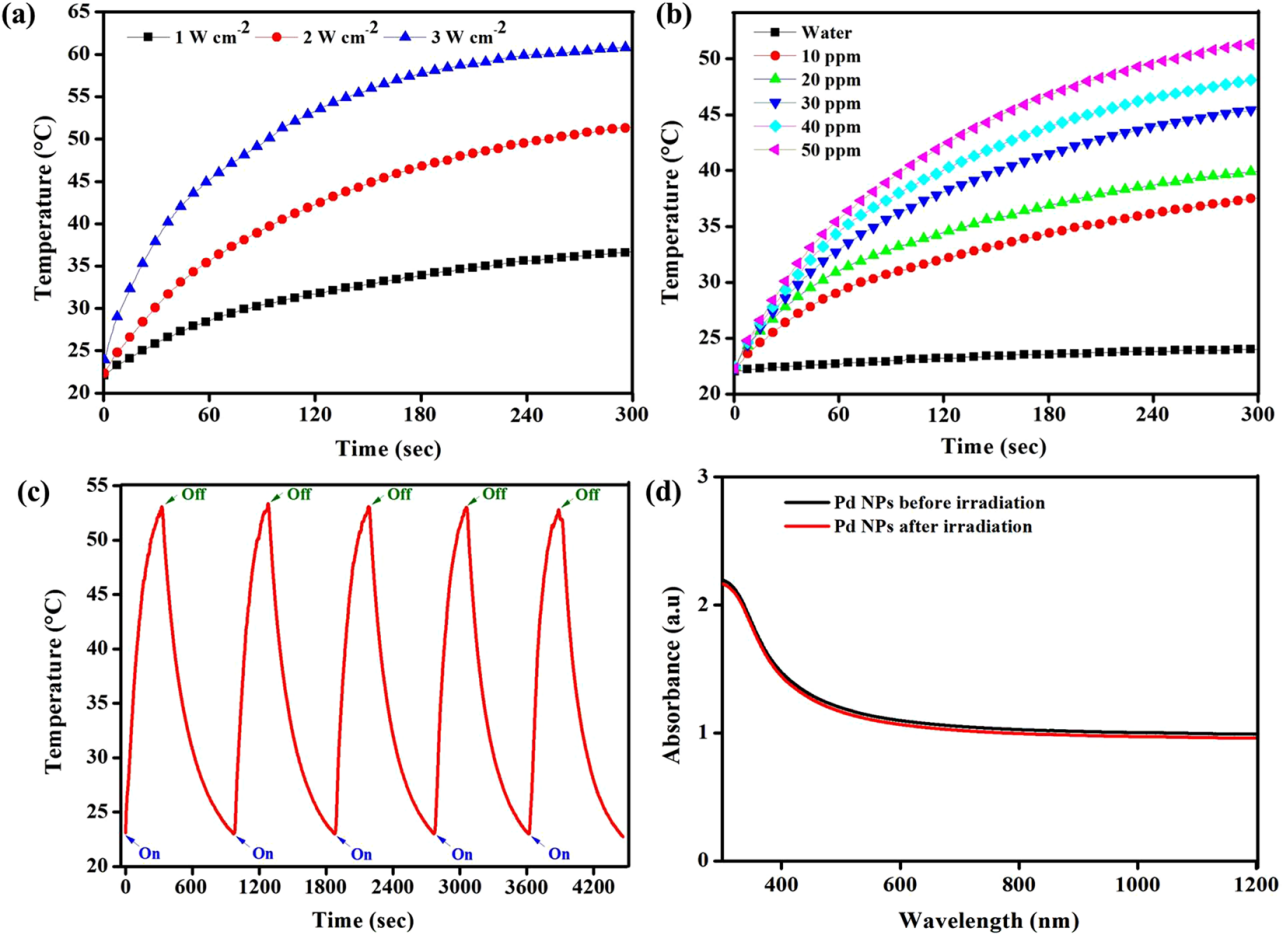
(**a**) Heating curve of Pd@COS-RGD (50 ppm) dispersed in 1 mL of water and irradiated with an 808-nm laser at different power densities. **(b)** Thermal curves of Pd@COS-RGD at different concentrations under 808-nm laser illumination at 2 W cm^−2^ power density. **(c)** Thermal stability of Pd@COS-RGD (50 ppm) over 5 cycles of a laser on/off experiment at 2 W cm^−2^ power density. **(d)** Optical absorbance spectrum of Pd@COS-RGD before and after 5 cycles of a laser on/off experiment.

The absorbance of Pd@COS-RGD falls between UV-Vis and NIR region which allows the use of different lasers. We selected an 808-nm laser for PTT treatment and PAT imaging applications. The NIR region range, from 700 to 1100 nm, has been described as a so-called transparency window for biological tissue and fluids^33^. Water has minimal absorbance at around 800 nm, which prevents the overheating of surrounding tissue regions^34^. As shown in Fig. 2a, Pd@COS-RGD has absorbance at 808 nm and the absorbance spectrum of Pd NPs is not affected by different surface modifications. Initially, the photothermal conversion efficiency of Pd@COS-RGD (50 ppm, 1 mL aqueous) was analyzed under 808-nm laser irradiation at different power densities. The initial environmental temperature was 22 °C, and this was kept as the initial temperature. Temperature elevations were recorded to be 36.8, 52.1, and 60.9 °C at 1, 2, and 3 W cm^−2^ power densities respectively (Fig. 3a). IR images captured during this experiment show the temperature distribution was even in the laser-irradiated 12-well plate (Fig. S4). A considerable amount of cell death can be achieved by exceeding the temperature 48 °C for few min. It is well known that cellular proteins tend to denature at temperatures in excess of 40 °C. Increasing the temperature beyond 42 °C causes cell inactivation and further temperature increases result in cell death^3^. The Pd@COS-RGD reached 42 °C in 2 min and 1 min at power densities of 1 and 2 W cm^−2^, respectively. At higher temperature around 60 °C and above for long time, the tissues could undergo explosion and tissue removal is produced due to water vaporization and other reasons^35^. Thus, we selected 2 W cm^−2^ as the optimum laser power density for further experiments. Then thermal response of Pd@COS-RGD at different concentrations at 2 W cm^−2^ is presented in Fig. 3b. The increase in the observed temperature was depended on the concentration of Pd@COS-RGD NPs. This temperature elevation was found to be 37.7, 40, 45.7, 48.3, and 53 °C at concentrations of 10, 20, 30, 40, and 50 ppm, respectively. Water without NPs did not exhibit a significant thermal response upon laser irradiation.

Laser on/off experiments were used to study the photostability of Pd NPs. An aqueous solution (1 mL) containing 50 ppm (Pd) of Pd NPs was irradiated for 5 cycles of power on/off experiment using 808-nm laser (2 W cm^−2^). For each cycle, the solution was irradiated for 5 min and the solution was allowed to cool down to room temperature naturally. The temperature changes recorded for the entire 5 cycles are presented in Fig. 3c. The Pd NPs displayed consistent thermal stability, reaching 53 °C after 5 min of laser irradiation for each repeated cycle. The absorbance spectrum of the Pd NPs was measured after the laser on/off experiment. The spectra of the Pd NPs solutions displayed no significant differences before and after irradiation (Fig. 3d). Compared with gold and silver nanostructures, Pd nanostructures have been reported to be the most stable photothermal transducers^7^.

The photothermal conversion efficiency of Pd NPs was compared with Au nanorods, as Au is the standard reference metal NPs. The optical density of both Pd NPs and Au nanorod solutions were adjusted to 1.0 absorbance unit at 808 nm. Then the solutions were irradiated with 808-nm laser at 2 W cm^−2^ power density for 5 min and the temperature was allowed to cool down naturally. The comparative thermal curve is presented in Fig. S5. As seen in Fig. S5, the thermal profiles of Pd NPs and Au nanorods were quite comparable. Huang at al. reported that Pd nanosheets serve as an efficient photothermal agent with 93.4% thermal conversion efficiency, which is comparable to Au nanorods^36^. Commonly used metal nanoparticles such as iron, gold, silver and platinum were extensively studied with different surface modifications, and some of them were used in clinical trials^37^. The number of studies on the use of Pd NPs as theranostic agents is scarce, and there is no study on the targeted accumulation of Pd NPs in tumor tissues.

### Biocompatibility of NPs

**Figure 4:**
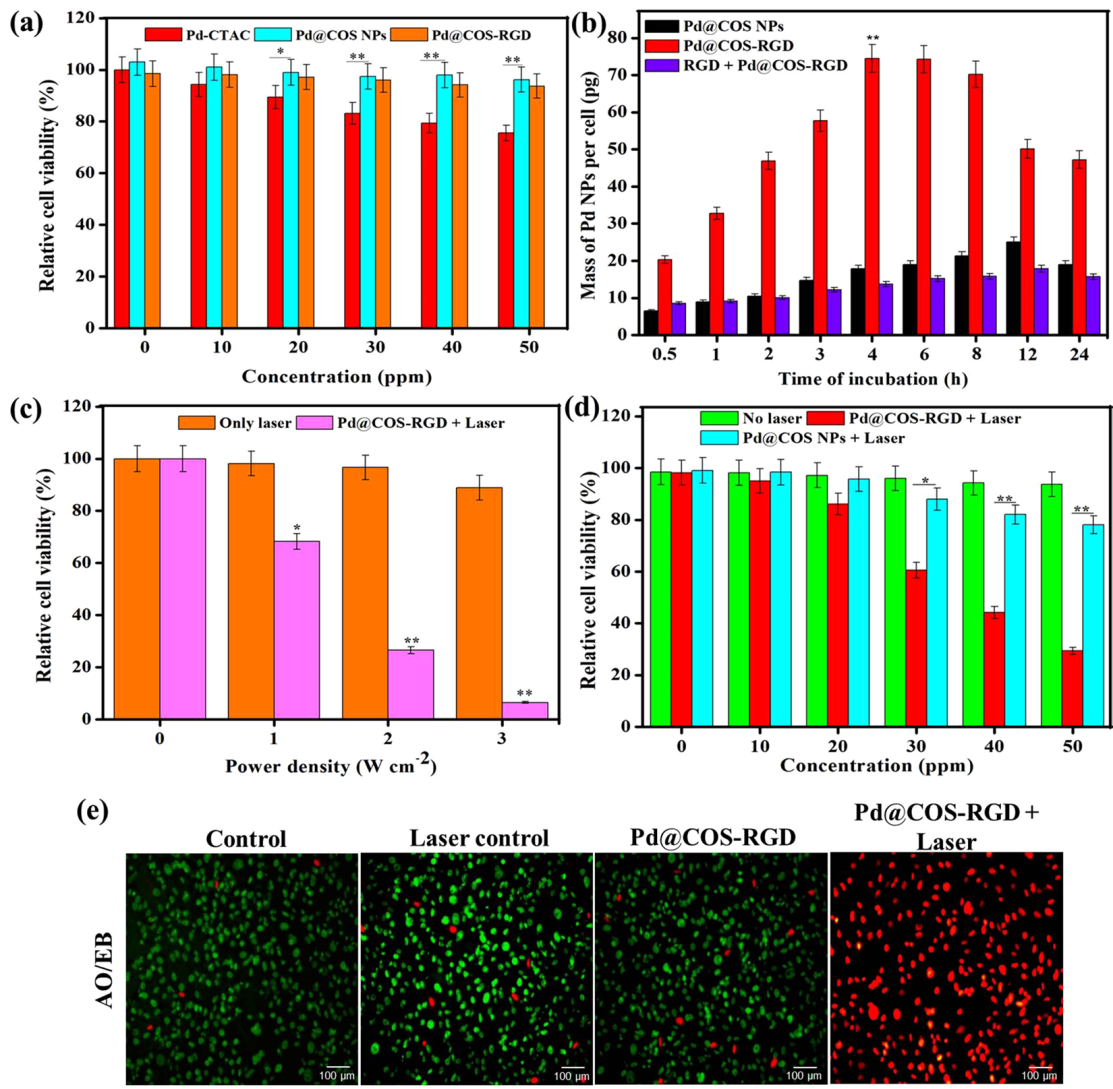
(**a**) *In vitro* cytotoxicity or biocompatibility of as-synthesized Pd NPs, COS-coated Pd NPs (Pd@COS NPs), and RGD-conjugated Pd@COS-RGD using the MTT assay. **(b)** Quantitative analysis of internalization of Pd@COS NPs and Pd@COS-RGD by MDA-MB-231 cells at different time intervals. **(c)** Viability of MDA-MB-231 cells incubated with or without Pd@COS-RGD (50 ppm) after 24 h of laser therapy using an 808-nm laser at different power densities. **(d)** *In vitro* photothermal cytotoxic effects of Pd@COS NPs and Pd@COS-RGD on MDA-MB-231 cells irradiated with 808-nm laser at 2 W cm^−2^ for 5 min. **(e)** Fluorescent images of MDA-MB-231 cells using AO/PI at different conditions and the concentration of Pd@COS-RGD fixed to be 50 ppm (green- live cells, red- dead cells). All data are presented as the mean ± standard deviation of three independent experiments (*p < 0.05, significant; **p < 0.01, highly significant).

The biocompatibilities of Pd NPs with different surface modifications were evaluated for 24 h using MDA-MB-231 cells. The concentration of each particle was fixed based on ICP-MS analysis of the Pd metal concentration. The results are shown in Fig. 4a, the Pd NPs without surface modification exhibited toxic effects toward MDA-MB-231 cells, resulting in 28% cell death at the concentration of 50 ppm. Since CTAC was used as a surfactant for the growth and stabilization of Pd NPs, it may have caused toxic effect to the cells. The surface-modified Pd@COS NPs reduced the toxicity of Pd NPs. No considerable cytotoxic effect was observed with concentrations ranging from 0 to 50 ppm (Fig. 4a). The biocompatibility of Pd@COS NPs was enhanced through the ligand exchange process that replaced CTAC with COS. Different studies have demonstrated the improved biocompatibility of COS-coated metallic NPs^38^. Similarly, Lin *et al*. observed better KB-cell biocompatibility with chitosan-coated copper NPs than with unmodified copper NPs^39^. The cell viability rate was slightly affected with Pd@COS-RGD, but no significant difference was observed when compared with Pd@COS NPs. Further, the biocompatibility of PD@COS-RGD was examined with different cell lines including human embryonic kidney cells (HEK-293) and human osteosarcoma cells (MG-63).The results are presented in Fig. S6 and shows that Pd@COS-RGD did not exert adverse toxic effects to the tested cell lines.

### Enhanced accumulation of Pd@COS-RGD

The cellular uptake of Pd@COS-RGD by MDA-MB-231 cells was examined using ICP-MS and flow cytometry (FACS) analyses. The uptake efficiencies in 4 h were found to be 74.06 ± 4.38 and 17.84 ± 2.13 pg for Pd@COS-RGD and Pd@COS NPs, respectively (Fig. 4b). Moreover, the uptake efficiency increased in a time-dependent manner up to 4 h, and the highest accumulation was measured at 4 h incubation. The cellular uptake of Pd@COS-RGD was greatly reduced when the MDA-MB-231 cells were pre-incubated with free RGD peptides, which block the integrin α_v_β_3_ cell-surface receptor. In this competitive assay, the accumulation of Pd@COS-RGD in RGD pre-incubated MDA-MB-231 cells after 4 h was found to be 13.43 ± 1.78 pg. The cellular uptake efficiency of Pd@COS-RGD was higher than that of Pd@COS NPs at each time interval, which represented integrin α_v_β_3_ receptor-mediated enhanced uptake of particles by MDA-MB-231 cells (Fig. 4b). Zhao *et al*. observed the same kind of cell-uptake efficiency using RGD-conjugated gold NPs in MDA-MB-231^24^. Further, the cellular uptake efficiency was investigated by FACS analysis using fluorescein isothiocyanate (FITC)-labeled particles. In this study, MDA-MB-231 cells were incubated with FITC-labeled Pd@COS NPs and Pd@COS-RGD for 1 h. Treated cells were collected and analyzed by FACS after 1 h of treatment. Fig. S7 shows that RGD conjugation enhanced the cellular uptake efficiency. The uptake efficiency was reduced when cells were pre-incubated with the free RGD peptide. The histogram of Pd@COS NPs shows that the efficiency of particle uptake was lower than that of Pd@COS-RGD. The result of FACS analysis was in agreement with the ICP-MS analysis of the study on cellular uptake.

### *In vitro* photothermal cytotoxicity

Prior to *in vivo* study, the photothermal cytotoxic effect of Pd@COS-RGD was evaluated in MDA-MB-231 cells using standard MTT assay. Initially, the photothermal toxicity of Pd@COS-RGD in MDA-MB-231 cells was assessed using 808-nm laser at different power densities. Figure 4C shows a drastic reduction in cell viability depends on laser power density. The cell death rate was recorded as 22.24, 73.8, and 94.1% at 1, 2, and 3 W cm^−2^ respectively. At the same time, an 11.7% cell death rate was observed in control cells treated at 3 W cm^−2^ power density. The thermal efficiency of Pd@COS-RGD NPs varied greatly depending on the power density of the 808-nm laser (Fig. 4c). Besides, cell death was less at 1 W cm^−2^ when compared to 2 W cm^−2^ with NPs treatment. Since the temperature reached 41 °C in 2 min and increased to 52 °C at 5 min (Fig. 3a), substantial cell death (73.32%) was observed at 2 W cm^−2^ power density. The 2 W cm^−2^ power density was chosen as optimum power density for further experiments. In this case, a drastic activation in cell death was achieved by increasing the temperature above 48 °C for a few min during treatment and it was considered as efficient and non-reversible cell death.

Pd@COS NPs and Pd@COS-RGD at the same concentration were incubated with MDA-MB-231 cells to compare the photothermal cell killing efficiencies of these particles. PTT was performed after 4 h of incubation with treated particles using 808-nm laser at 2 W cm^−2^. The MTT assay was performed after 24 h of laser treatment. Figure 4d shows the effective cell death in Pd@COS-RGD treated cells, which was significantly higher than that of Pd@COS NPs treated cells. This can be explained by a specific interaction between Pd@COS-RGD and MDA-MB-231 cells that result in higher accumulation of NPs in the cells. *in vitro* PTT showed higher accumulation of Pd@COS-RGD than of Pd@COS NPs to MDA-MB-231 cells. The cell death rate increased depending on the concentration of Pd@COS-RGD. The viability of cells found to be 95.4, 82.4, 59.9, 45.6, and 26.2% at 10, 20, 30, 40, and 50 ppm respectively. The viability of control cell without particles was not affected. The results demonstrated that laser at 2 W cm^−2^ did not have an obvious effect on cell viability. AO/PI staining was carried out to visually demonstrate cell death. Figure 4e demonstrates that almost all of the cells in the control group were alive (green color) with or without laser irradiation. Only a negligible number of dead cells (red color) was observed in the laser control and Pd@COS-RGD incubated cells. However, when the Pd@COS-RGD incubated cells were exposed to laser irradiation, most of the cells died. The bright-field images also demonstrate the disturbed cell death morphology in Pd@COS-RGD incubated, laser-treated cells (Fig. S8a). The morphology of the cells was uniform and undisturbed in the control, laser control and Pd@COS-RGD treated cells. The Pd@COS-RGD + laser treated cells were lysed and the morphology of the cells was highly altered (Fig. S8a).

PTT-induced apoptotic cell death was further examined by flow cytometry after 8 h of laser treatment. The PTT-treated cells were double stained with FITC-Annexin V and PI, and then FACS analysis was performed. The results revealed that a large portion of the Pd@COS-RGD + laser-exposed cells had undergone early apoptosis (49.1%); simultaneously, some cells had progressed to the late apoptosis/necrosis phase (12.5%) (Fig. S8b). We recognized that most of the cells in the control, laser control, and NPs control groups (about 98.4, 92.7, and 95.2% respectively) were plotted in the live state (Fig. S8b). Therefore, Pd@COS-RGD exerted obvious damage to MDA-MB-231 cells when irradiated with 808-nm laser light at 2 W cm^−2^. Altogether, the results of *in vitro* study suggest that the Pd@COS-RGD possess great potential for NIR activated photothermal toxicity through its enhanced accumulation into MDA-MB-231 cells.

### *In vivo* Biodistribution

**Figure 5:**
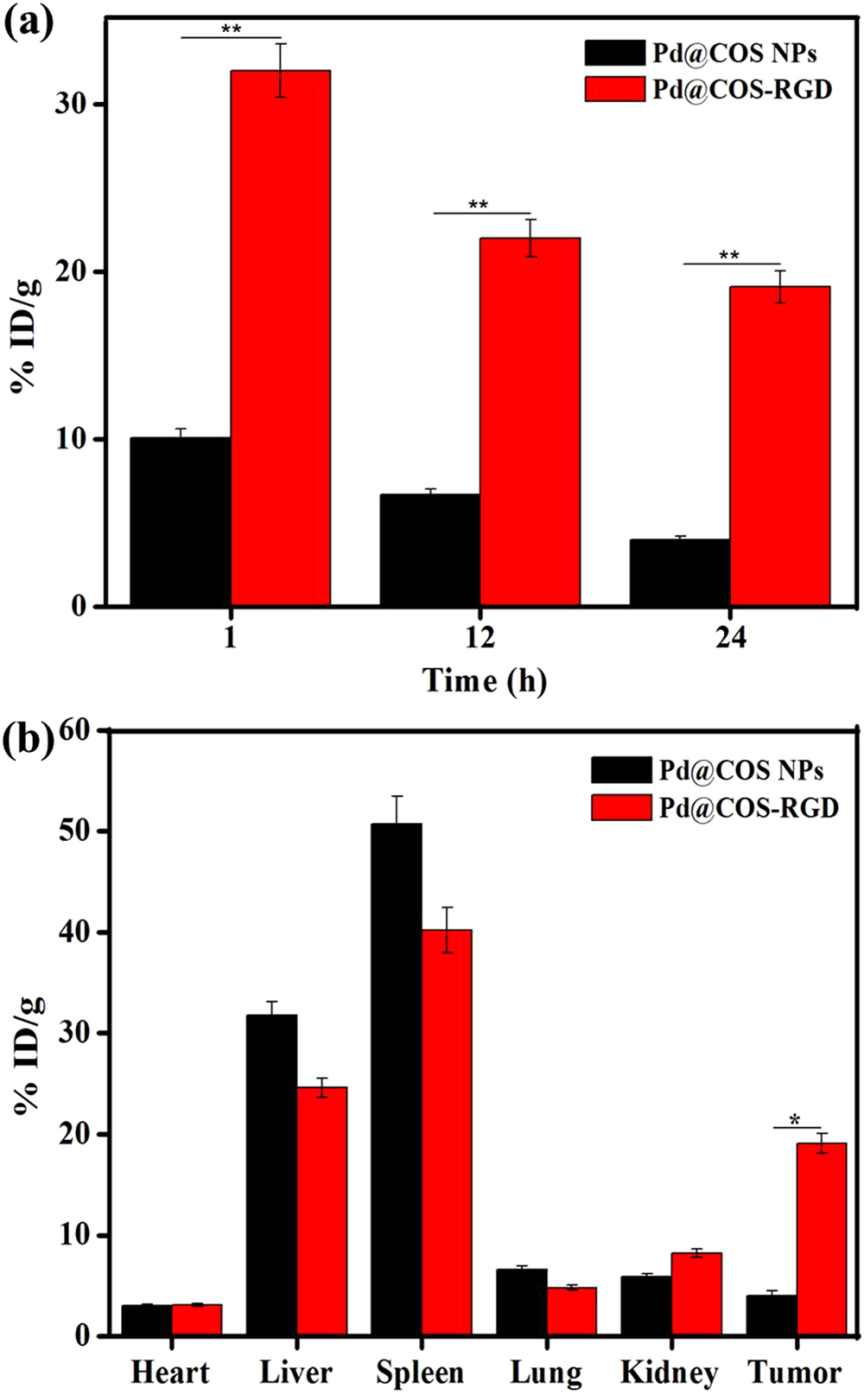
(**a**) *In vivo* tumor accumulation of Pd@COS NPs and Pd@COS-RGD at different time intervals. **(b)** Biodistribution of Pd@COS NPs and Pd@COS-RGD in major mice organs and tumor after 24 h of intravenous injection. The Pd metal concentration was analyzed using ICP-MS. The data are presented as the mean ± standard deviation (*p < 0.05, significant).

To explore *in vivo* tumor accumulation, Pd@COS NPs and Pd@COS-RGD were delivered to the MDA-MB-231 tumor established mouse via tail vein injection. The tumor of the mice were dissected at 1, 12, and 24 h post the injection of particles and the tumor tissue was lysed with aqua regia to measure the Pd particles by ICP-MS analysis. Figure 5a shows that RGD-functionalized Pd@COS-RGD was homed in the tumor highly than Pd@COS NPs at all time intervals. The higher accumulation rate occurred at 1 h after the tail vein injection.

Then *in vivo* biodistribution of the Pd@COS NPs and Pd@COS-RGD was examined in major organs of mice after 24 h of intravenous injection. The concentration of Pd was higher in the tumor tissues of mice that received a Pd@COS-RGD injection than in those of mice treated with Pd@COS NPs (Fig. 5b). Figure 5b shows that a large amount of Pd@COS NPs/Pd@COS-RGD detected in spleen and liver after 24 h of injection, which shows that both NPs were eliminated through the reticuloendothelial system. It is well documented that different parameters such as size, shape, and surface chemistry influence the renal clearance of NPs in the *in vivo* system^40^. A nearly 4-fold higher amount of Pd was detected in the tumor tissues of Pd@COS-RGD-treated mice even 24 h after injection (Fig. 5b). This may be the reason behind the lesser accumulation of Pd in the liver and spleen of Pd@COS-RGD-treated mice than in that of Pd@COS NPs treated mice. The Pd@COS NPs could have passively accumulated to the tumor site. It is interesting to note that the accumulation of Pd was significantly increased by RDG peptide conjugation, which enables active targeting of tumor tissue. Several research groups have reported the higher accumulation of RGD peptide-conjugated NPs in the MDA-MB-231 tumor model^24,34^. By considering the results of the *in vitro* cell study and the *in vivo* tumor accumulation rate, Pd@COS-RGD was advanced to *in vivo* tumor imaging and phototherapy applications using NIR laser.

### *In vivo* PTT

**Figure 6:**
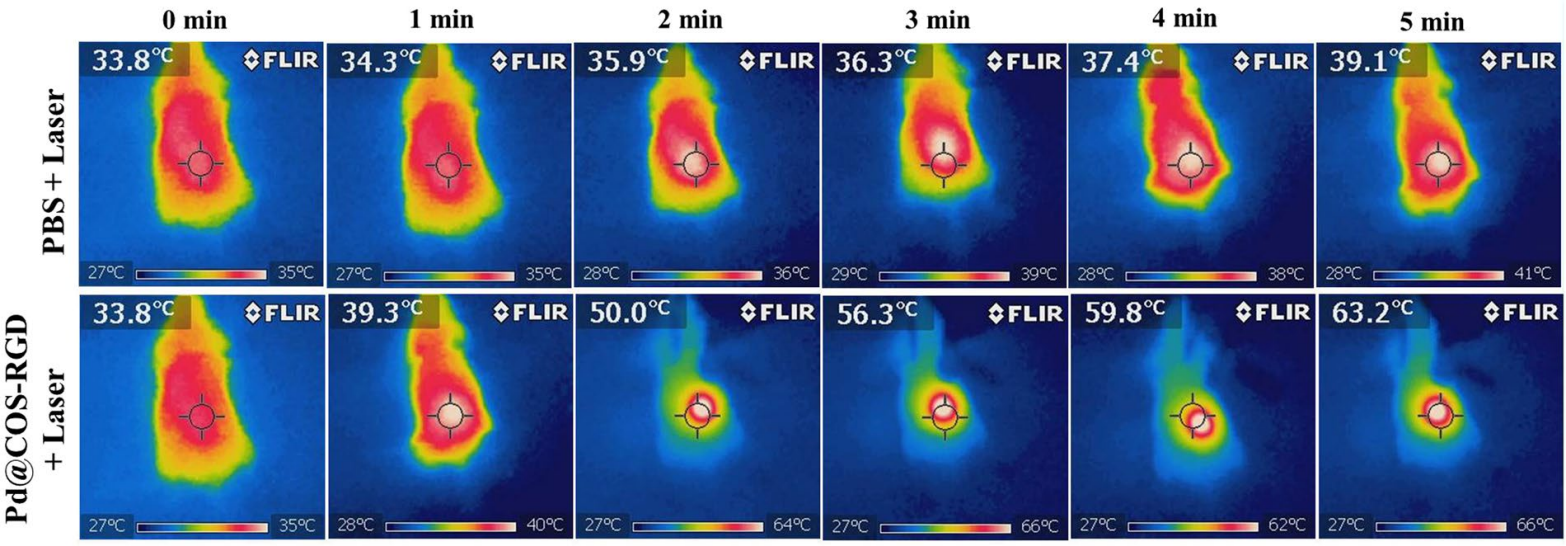
Infrared thermograph of MDA-MB-231 breast tumor-bearing nude mice irradiated with 808-nm laser at 2 W cm^−2^ after 1 h of intravenous injection of PBS and Pd@COS-RGD.

**Figure 7:**
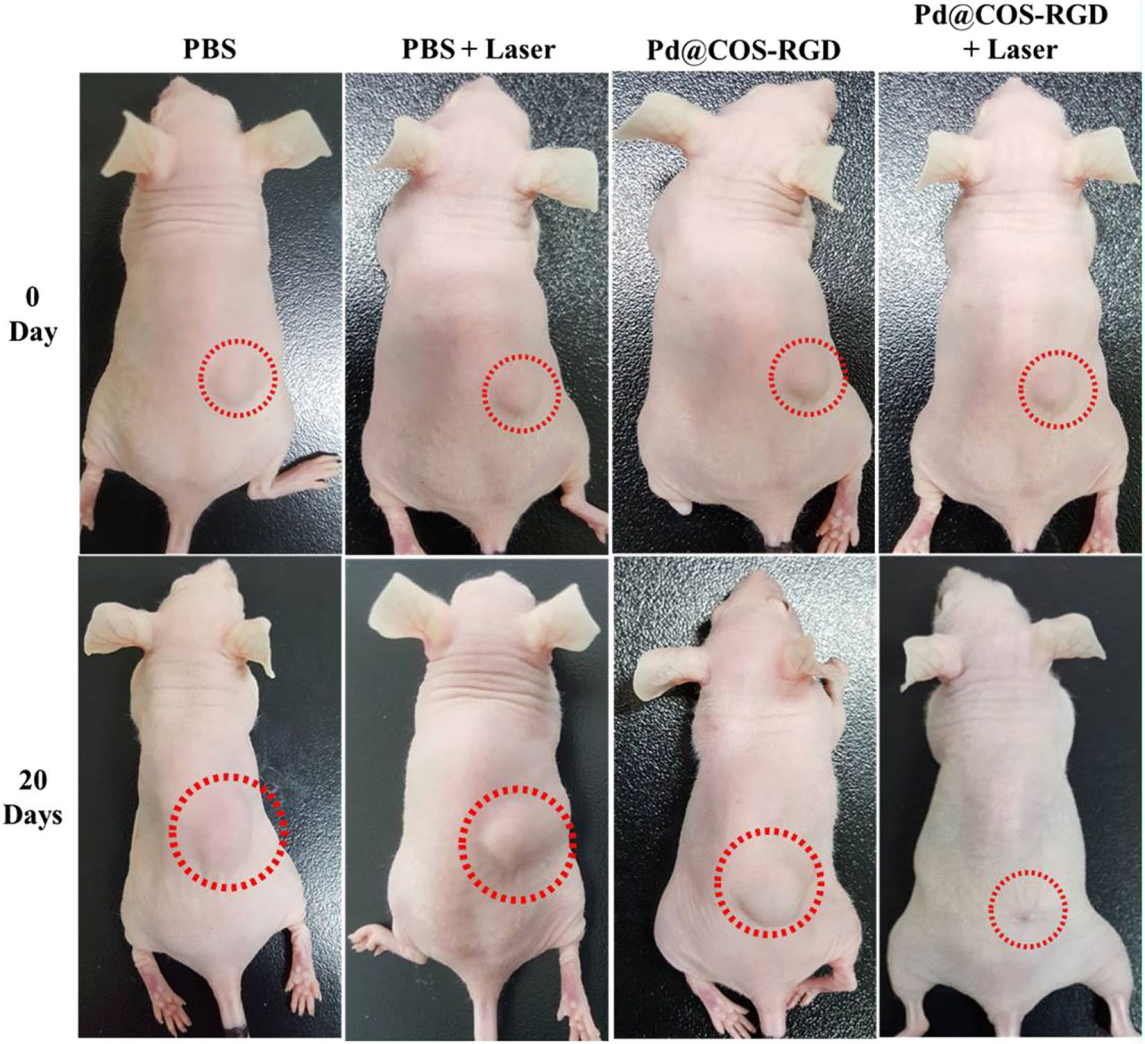
Photograph of MDA-MB-231 tumor-bearing mouse on 0 and on the 20^th^ day of observation after intravenous injection of PBS and Pd@COS-RGD (5 mg/kg). The 808-nm laser irradiated the tumor region for 5 min at 2 W cm^−2^ power density after 1 h of intravenous injection.

**Figure 8:**
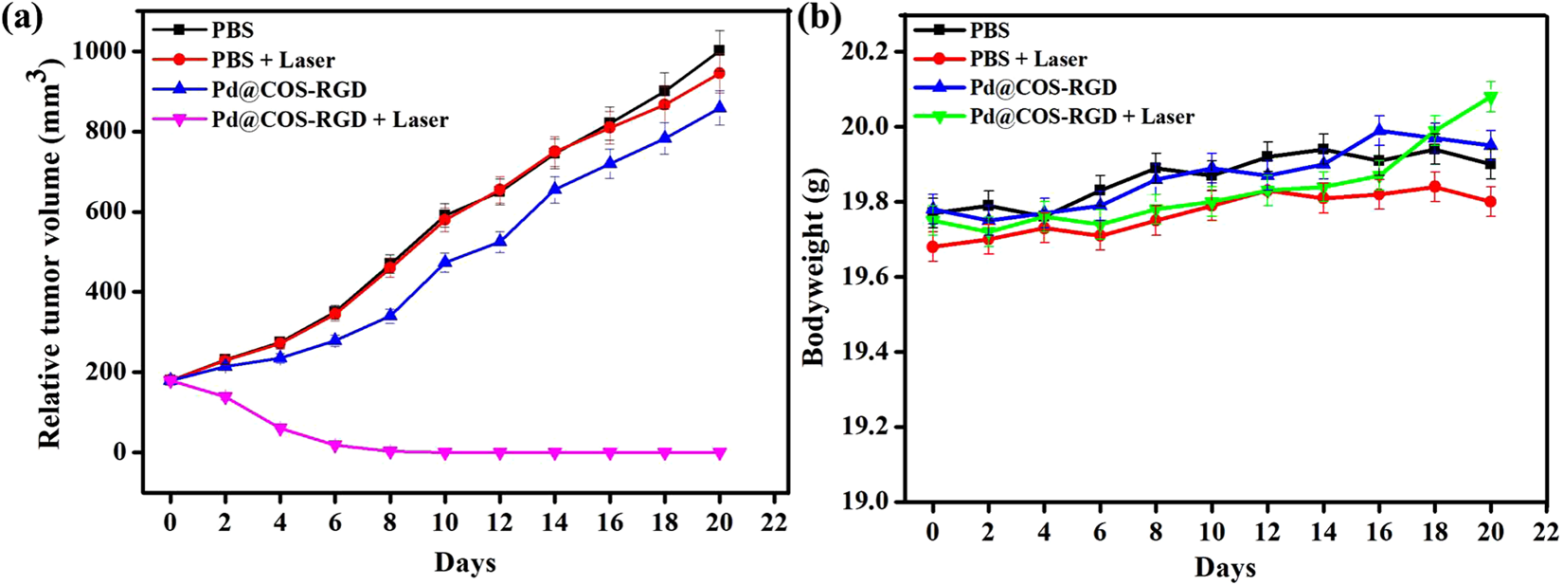
(**a**) Tumor growth curves of different groups of MDA-MB-231 tumor-bearing mice normalized against initial tumor volume. **(b)** The mean body weight of different groups of animals over 20 days after treatment. Data are presented as the mean ± standard deviation.

Following *in vitro* evaluation, we performed *in vivo* PTT in BALB/c nude mice bearing MDA-MB-231 xenograft tumor. When the tumor size reached about 170–180 mm^3^, the animals were divided into four groups based on treatment type as follows: PBS; PBS + laser; Pd@COS-RGD; and Pd@COS-RGD + laser. After laser treatment, the tumor suppression and progression rate were measured for up to 20 days. The surface temperature change in the tumor region during laser exposure is depictured in Fig. 6. The tumor temperature in the Pd@COS-RGD + laser group reached 63.2 °C in 5 min, whereas the temperature was found to be 39 °C in PBS + laser group. Pd@COS NPs treated mice were irradiated under laser for comparatively analyzing the thermograph and confirming the *in vivo* tumor accumulation of particles. Fig. S9a shows that the temperature reached 45.8 °C in Pd@COS NPs treated mice at the end of laser therapy. The comparative thermal elevation is presented in Fig. S9b. The study implied that RGD-functionalized Pd@COS-RGD accumulated more in the tumor region than Pd@COS NPs and resulted in the enhanced effects of laser therapy. The temperature reached to 50 °C during the 2^nd^ min of laser exposure in Pd@COS-RGD + laser treated mice, which is enough to kill the cells^3^. The tumor sizes in the different animal groups were tested every 2 days after laser treatment. The photograph of *in vivo* PTT, depicted in Fig. 7 that showed complete disappearance of the tumor in Pd@COS-RGD + laser group on the 20^th^ day. The thermal destructed tumor was reduced and healed completely on the 20^th^ day (Fig. S10). An increased tumor size was observed in the remaining animal groups (Fig. 7). The tumor sizes of the PBS injection group (group I), the PBS plus laser irradiation group (group II), and the Pd@COS-RGD injection without laser irradiation group (group III) were rapidly increased to 1001.66, 942.71, and 857.68 mm^3^, respectively, on the 20^th^ day (Fig. 8a). The tumor volume almost disappeared in group IV animal which is treated with 808-nm laser irradiation after 1 h of Pd@COS-RGD delivery via tail vein injection. The tumor volume was reduced to 60.84 mm^3^ on 4^th^ day after treatment. The tumor volume began to decrease from the first day after laser treatment, and the size became undetectable on the 8^th^ day after treatment. The Pd@COS-RGD treated mice (group III) showed a slight reduction of tumor mass, while PBS (group I) and PBS + laser (group II) groups did not show any tumor suppression (Fig. 8a). As shown in Fig. S11, on the 20^th^ day, the tumor was relapsed and undetectable in Pd@COS-RGD + laser group. No significant regression of tumor weight was observed in the other groups (I, II or III; Fig. S11). These results imply that Pd@COS-RGD exhibited remarkable *in vivo* PTT efficacy. Moreover, neither death nor significant body weight variance was detected in any animal group during the experimental period, which implies the reliable biosafety of Pd@COS-RGD *in vivo* (Fig. 8b). Different concentrations of Pd@COS-RGD were injected to mice to screen for the toxic effects of Pd. Animal behavior and bodyweight were observed for 20 days. The results show that the particles did not induce any adverse side effect till a concentration of 200 µg was reached (Fig. S12). Significant body weight loss was observed in the animal group that was injected with 400 µg of Pd@COS-RGD(Fig. S12). Overall, this *in vivo* study implied that Pd@COS-RGD was safe and did not cause any side effects or death at the fixed concentration.

To understand the clearance or bioremoval of the particles, the Pd@COS-RGD injected mice were sacrificed after 20 days of the experiment. Their major organs and tumor were removed and solubilized in aqua regia for measurement of Pd using ICP-AES. The results were compared with the 24-h biodistribution data and the comparison is presented in Fig. S13. The ICP-MS analysis showed a great reduction in Pd on the 20^th^ day in group IV animals, compared with the 1^st^ day after injection of Pd@COS-RGD particles. The substantial accumulation of Pd in the RES system remained over 20 days after injection, and it was significantly lower that observed on the1^st^ day after injection. The tumor of Pd@COS-RGD + laser treated mice completely disappeared on the 20^th^ day post treatment, and no trace of Pd was detected in the cured region.

### Histology examination

The histological images of tissues presented in Fig. S14. The major organs were excised out to evaluate the toxic effects of Pd@COS-RGD and laser application in different groups of animals. All of the major organs (heart, liver, spleen, lung, and kidney) appeared to be normal in all the treatment groups. No considerable changes were observed among the treatment groups. The lack of tissue damage, abnormal behavior, and body weight changes suggests the overall nontoxic nature of Pd@COS-RGD. The histology of tumor tissues also presented in Fig. S14. Moderately and poorly differentiated tumors with granulated nuclei were observed in the PBS, PBS + laser, and Pd@COS-RGD animal groups. Pd@COS-RGD + laser treated animals showed recovered tissue containing fewer granulated nuclei.

### PAT imaging

**Figure 9:**
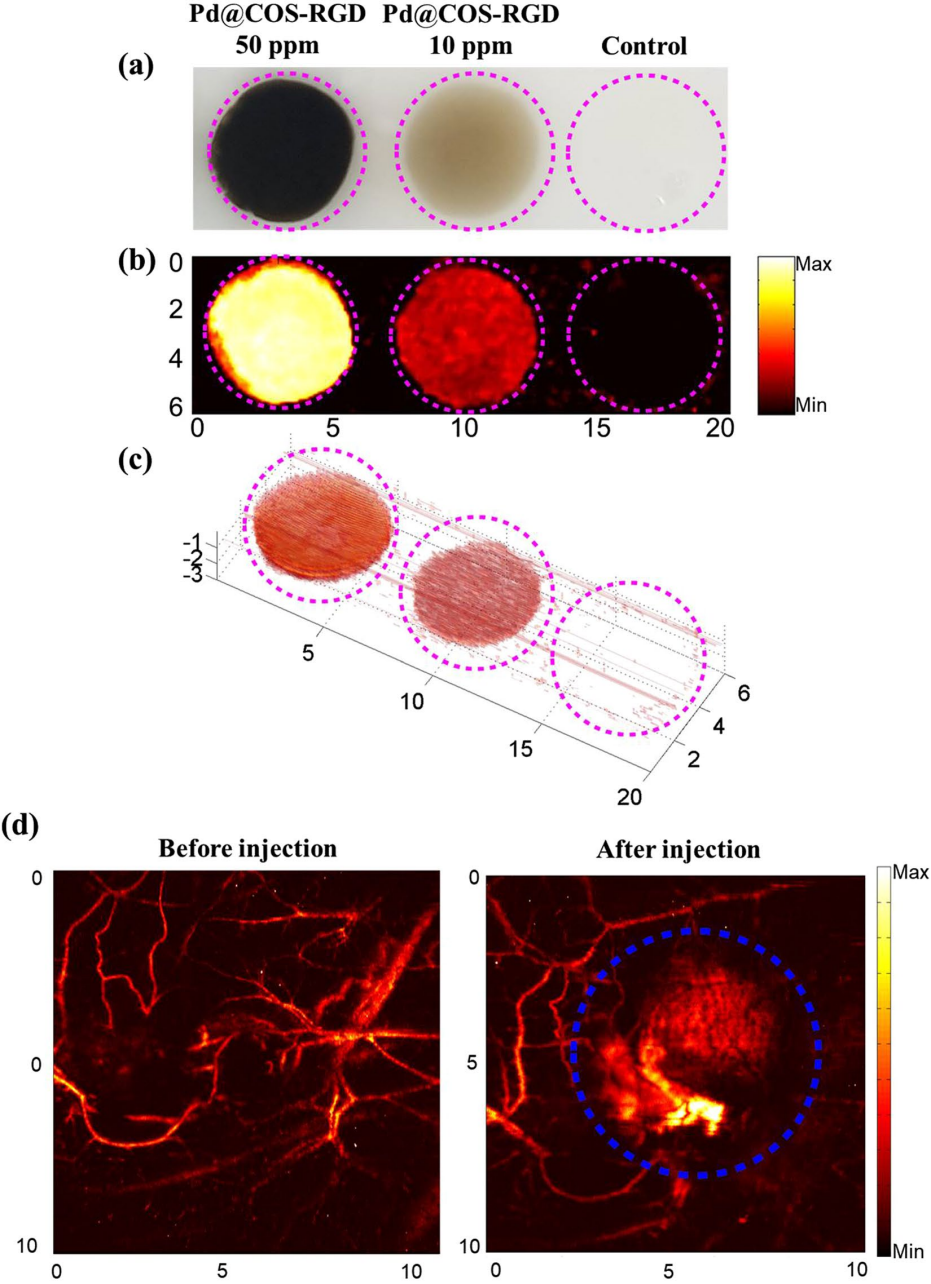
(**a**) Top view of tissue-mimicking phantom loaded with Pd@COS-RGD treated and untreated control MDA-MB-231 cell inclusions. *in vitro* photoacoustic imaging of control and treated cells using 808-nm laser represented with 2D **(b)** and 3D images **(c)**. **(d)** *In vivo* photoacoustic imaging of MDA-MB-231 tumor-bearing mice before and after 1 h of tail vein injection of Pd@COS-RGD.

PAT imaging is emerging as a non-invasive biomedical imaging technology that combines optical contrast and ultrasound-based signals to image the treated tissue. NIR-absorbing NPs are able to generate photoacoustic signals (PA) inside the tissue by thermal expansion upon NIR laser illumination^41^. NIR-absorbing NPs can act as external contrast agents for PAT applications and their use has been manifested in biomedical imaging applications. Endogenous chromospheres like hemoglobin and melanin will not differentiate diseased tissues from normal tissues. Thus, the development of exogenous PAT contrast agents facilitates the imaging of specific tissues for diagnosis. It also helps to monitor the progress of treatment. Owing to the good photothermal effects of Pd, we studied the PAT efficiency of Pd@COS-RGD *in vitro* and then *in vivo*. A human tissue-mimicking phantom was prepared for the *in vitro* PAT study. MDA-MB-231 cells were treated with Pd@COS-RGD (10 and 50 ppm) for 4 h, then cells were loaded in the phantom with 8% gelatin, while untreated cells were considered as a control. Figure 9a shows the top view of the tissue-mimicking phantom loaded with cells under different conditions. The cells labeled with Pd@COS-RGD exhibit PA signal amplitude which distinguishes these cells from the background of the phantom. The PA signal amplitude increased with the concentration of NPs, and it was not detected in the control group because cells themselves do not absorb NIR light. The *in vitro* 2D images clearly showed the tissue-like cell clumps in the phantom (Fig. 9b). The 3D PAT image, constructed using a home-made Matlab program (Version: 2013, MathWorks, Natick, MA, USA), is presented in Fig. 9c. This shows a 3D view of a tissue-like cell clump inside the phantom.

Owing to the excellence of the PA signals and imaging efficiency from the *in vitro* study, we moved to examine the effect of Pd@COS-RGD *In vivo*. *in vivo* PAT was performed before and after tail vein injection of Pd@COS-RGD into the mice. Mouse injected with PBS was used as a control and the same mouse injected with Pd@COS-RGD was considered as a treated animal. The scanning area was fixed to be same before and after injection of particles into the mice. The PAT experiments were performed after 1 h of intravenous injection. The results are presented in Fig. 9d. It was clear that the injected Pd@COS-RGD accumulated in the tumor region and generated the given image of the tumor tissue. Pd@COS-RGD acted as an excellent contrast agent, generating PA signals that produced an image of the treated tissue. In the control, only the blood vessels were observed and no evidence of tissue was detected. The high amplitude PA signals detected in treated mouse and the same was absent in control mouse. The RGD-conjugated nanoprobe possesses active targeting ability, which helps to identify cancer tissue easliy^42^. The utilization of Pd NPs for PAT has not been explored much, except by Chen *et al*. who analyzed the size-dependent PA imaging effect of Pd NPs^16^.

Significant challenges still remain in advancing the use of photothermal nanoparticles from laboratory settings to clinical therapy and further struggles to obtaining approval from Food and Drug Administration. Concern needs on reproducibility and uniformity of nanoparticles when production is scaled up^43^. Even though different nanoparticles have been reported to be biocompatible and for the targeted delivery for precise photothermal therapy, no investigation is present on the targeted delivery of biocompatible Pd NPs. In the present study, nanocomposites composed of chitosan oligosaccharide, palladium nanoparticles and RGD peptide were successfully fabricated for targeted photothermal therapy and photoacoustic imaging of tumor. The investigation revealed the potential of palladium in image-combined phototherapy and its possible utilization in biomedical fields. The photothermal transduction efficiency of palladium was comparable with that of gold particles and it had the highest photothermal-optical stability. The formulated particles were biocompatible and showed good physiological stabilities. The particles were efficiently homed into the tumor site. This study proved the enhanced internalization of synthesized particles leading to efficient PTT combined with photoacoustic imaging at the *in vitro* and *in vivo* levels. We believe that chitosan oligosaccharide coated Pd NPs have the highest potential for cancer-targeting functionalization, which encourages further exploration of these particles in biomedical fields.

## Methods

### Synthesis of porous Pd NPs

Porous Pd NPs were synthesized by adapting the protocol of Wang *et al*.^25^. Briefly, 0.01 M aqueous H_2_PdCl_4_ was prepared by dissolving 44.5 mg of PdCl_2_ in 25 mL of 0.02 M HCL at 50 °C. Then, 0.25 mL of this H_2_PdCl_4_ solution was mixed with cetyltrimethylammonium chloride (CTAC) (0.1 M, 9.75 mL). To this reaction mixture, freshly prepared ice-cold NaBH_4_ (0.01 M, 0.60 mL) was added with vigorous stirring and the reaction solution was kept undisturbed for 3 h at room temperature as a seed solution. For the growth of Pd NPs, 0.025 mL of seed solution was added to a mixture of CTAC (4.5 mM, 9.70 mL), H_2_PdCl_4_ (0.01 M, 0.30 mL), and ascorbic acid (0.1 M, 0.1 mL). The reaction mixture was mixed by gentle inversion for 10 s and left undisturbed at room temperature for 6 h. The resulting Pd NPs were collected by centrifugation at 20000 *g* for 5 min.

### Modification of COS

COS was modified with thiol groups by following the protocol by Anitha *et al*.^44^. with some modifications^44^. TGA was used to modify COS to obtain the thiol (-SH) groups containing COS (COS-SH), which can interact with Pd NPs. TGA was conjugated with the amine part of COS units after the activation of carboxylic acid groups present in TGA using EDC. First, approximately 500 mg of TGA was dissolved in 1 mL of distilled water. To this, approximately 125 mM of EDC was added, and the reaction mixture was stirred for 6 h at RT. Next, activated TGA was slowly added to the reaction flask containing 500 mg of COS dissolved in 50 mL of 0.1% acetic acid. The reaction mixture was stirred for 4 h, and then, thiolated COS (COS-SH) was purified by dialysis using a 3.5-kDa cellulose membrane. Finally, the product was lyophilized for further studies.

### Surface modification of Pd NPs with COS-SH

For this modification, approximately 20 µL (500 mg/mL) of COS-SH solution was slowly added to the reaction flask containing Pd NPs (10 mL, 100 µg/mL) under sonication, and the resulting mixture was then magnetically stirred overnight (Fig. S1, step-1). The obtained COS-coated Pd NPs (Pd@COS NPs) were collected by centrifugation at 18,000 rpm for 15 min. Step-1 was repeated three times to achieve maximum surface coating of Pd NPs. The free amino group content of Pd@COS NPs was calculated using 2,4,6-trinitrobenzenesulfonic acid (the TNBS) method. The initial absorbance of Pd@COS NPs at 345 nm was subtracted from the absorbance of Pd@COS Nps + TNBS assay and COS in different concentrations was kept as control.

### Formulation of Pd@COS-RGD

The thiol group containing peptide c-(RGDfK)-SH (RGD peptide) was covalently conjugated with the Pd@COS NPs using the “thiol-ene” click chemistry approach^45^. To achieve this, first, Pd@COS NPs were conjugated with maleic anhydride groups via ring opening reaction approaches shown in Fig. S1 (step-2). To perform this step, approximately 2 mL (0.01 M) of maleic anhydride was added to the reaction flask containing Pd@COS NPs dispersed in dry ethanol (10 mg/mL, 10 mL). Next, the RGD peptide was conjugated with Pd@COS NPs via reaction between the thiol (-SH) groups of the RGD peptide units and the ene (C=C) part of maleic anhydride, which coupled onto the outer surface of Pd@COS NPs (Fig. S1, step-3). For this conjugation, the maleic anhydride-modified Pd@COS NPs (10 mg/mL) were dispersed in 10 mL of distilled water using ultrasonication. To this, a solution of RGD peptides (1 mg/mL) was added drop wise, and the resulting suspension was kept at room temperature for 2 h in the presence of catalytic trimethylamine base. The RGD peptide-conjugated Pd@COS NPs were then separated by centrifugation (12,000 rpm, for 10 min). The unreacted RGD peptide in the supernatant was measured with a BCA kit by measuring absorbance at 562 nm29. The RGD peptide-conjugated sample was designated as Pd@COS-RGD (Fig. S1, step-3).

### Characterization

UV-Vis-NIR spectra of the particles were obtained using quartz cuvette with a 1-cm light path using a UV-Vis spectrophotometer (Beckman Coulter, Fullerton, CA, USA). The images of particles during synthesis and modification were captured under transmission electron microscope (JEM 1010 JEOL, Tokyo, Japan) by placing aliquots of aqueous particle suspension on carbon-enhanced copper grids and accelerating the machine at 200 kV. The size distribution of the particles was analyzed using a Zeta instrument (ELS-8000, OTSUKA Electronics Co. Ltd., Japan). The X-ray diffraction (XRD) spectra of the particles were measured using XRD (X’Pert-MPD, Philips, Amsterdam, The Netherlands) with Cu-Kα radiation. To analyze the functional groups of the particles at different stages, aliquots of freeze-dried product were mixed with KBr and pelletized for FTIR analysis (Perkin Elmer Inc., Waltham, Massachusetts, USA) at a resolution of 4 cm^−1^ over the wavelength range 500 to 4000 cm^−1^. Thermogravimetric analysis was performed using TGA 7, Pyris 1 (Perkin Elmer, MA, USA).

### NIR thermal experiments

Pd@COS-RGD were prepared at Pd concentration of 50 ppm of (measured by ICP-MS) and brought to a final volume of 1 mL per well in a 12-well plate. Each sample was irradiated under an 808-nm NIR laser for 5 min (Changchun New Industries Optoelectronics Technology Co., Ltd., Changchun, China) at different power densities (1, 2, and 3 W cm^−2^). The temperature was monitored each second using a thermo fiber. Further, different concentrations of Pd@COS-RGD (10, 20, 30, 40, and 50 ppm) in 1 mL of aqueous solution were irradiated under an 808-nm laser at 2 W cm^−2^ power density. The thermal stability of the Pd NPs was analyzed with laser on/off experiment at 2 W cm^−2^ power density using an 808-nm laser for 5 cycles. The temperature was allowed to cool down naturally during the laser on/off experiment. The UV-Vis absorbance spectrum of the irradiated solution was measured after 5 cycles of the on/off experiment.

### Cell culture

MDA-MB-231 human breast cancer cells, HEK-293 and MG-63 cells were cultured and maintained in Dulbecco’s Modified Eagle’s Medium (DMEM) containing 10% FBS supplemented with 100 U mL^−1^ penicillin and 100 µg mL^−1^ streptomycin. The cultures were maintained in a CO_2_ incubator at 37 °C in a humidified atmosphere.

### *In vitro* biocompatibility test

MDA-MB-231 cells (1 × 10^4^ per well) were seeded in 96 well plates. After 24 h, the cells were treated with a series of concentrations (10, 20, 30, 40, and 50 ppm) of as-synthesized Pd NPs, a similar series of COS-modified Pd@COS NPs, or a similar series of RGD-functionalized Pd@COS-RGD for 24 h. Then, the cells were incubated with 100 µL of 3-(4,5-dimethylthiazol-2-yl)-2,5-diphenyltetrazolium bromide (MTT) (0.5 mg mL^−1^) for 3 to 4 h. After incubation, the standard thiazolyl tetrazolium assay was performed to examine relative cell viability^46^. In addition, HEK-293 and MG-63 cells were utilized to screen the biocompatibility of Pd@COS-RGD as mentioned above.

### Cellular uptake

MDA-MB-231 cells were seeded onto 6-cm Petri dishes at a concentration of 1 × 10^6^ cells per plate. After the cells reached 70–80% confluence, Pd@COS NPS and Pd@COS-RGD were added separately at a concentration of 50 ppm Pd. To confirm RGD-mediated cellular uptake, free RGD peptide was added to cells to block the receptor before adding Pd@COS-RGD in a separate assay. Then, the cells were incubated for different time periods (0.5, 1, 2, 3, 4, 6, 8, 12, and 24 h). At the end of each time period, cells were collected, resuspended in PBS, and adjusted to a final volume of 5 mL. Then, the cells were counted using a hemocytometer and lysed in aqua regia. The amount of Pd was measured using inductively coupled plasma atomic emission spectroscopy. The amount of Pd internalized by the cells was calculated using the following formula.

$${\rm{Mass}}\,{\rm{of}}\,{\rm{Pd}}\,{\rm{per}}\,{\rm{cell}}=\frac{{\rm{mass}}\,{\rm{of}}\,{\rm{Pd}}\,{\rm{in}}\,{\rm{the}}\,{\rm{cell}}\,{\rm{lysis}}}{{\rm{number}}\,{\rm{of}}\,{\rm{cells}}}$$

Additionally, FACS analysis was performed with FITC-labeled Pd@COS NPs and Pd@COS-RGD for cell uptake analysis. The conjugation of FITC with the free amine groups of COS was done according to the protocol by Haung *et al*.^47^. MDA-MB-231 cells were cultured and incubated with FITC-labelled Pd@COS NPs and Pd@COS-RGD for 1 h. In another assay, cells were pre-incubated with the free RGD peptide and then incubated with Pd@COS-RGD for 1 h. After 1 h of incubation, the cells were collected and resuspended in PBS and then analysed by flow cytometry (BD Biosciences, CA, USA). The concentration and fluorescent intensity of the particles were fixed to be same before they were treated with cells.

### *In vitro* photothermal cytotoxic assay

To assess the photothermal effect of Pd@COS-RGD in MDA-MB-231 cancer cells, the cells were seeded in 96-well plates at a density of 1 × 10^4^ cells per well and treated with media containing formulated Pd@COS-RGD particles at concentrations of 10, 20, 30, 40, and 50 ppm of Pd. Cells without nanoparticles were maintained as controls. After 4 h of incubation, the media was replaced with fresh media and allowed to stand for 30 min. Then, the cells were irradiated with an 808-nm laser at the power density of 2 W cm^−2^ for 5 min. The photo-illuminated cells were incubated for an additional 24 h, and then screened for cell viability using the MTT assay. In addition, MDA-MB-231 cells were separately treated with 50 ppm of Pd@COS-RGD and irradiated with an 808-nm laser at different power densities (1, 2, and 3 W cm^−2^) to screen the PTT cytotoxicity.

### Staining of irradiated cells

Acridine orange (AO) and propidium iodide (PI) fluorescent stains were used to visualize the PTT-induced cell death in Pd@COS-RGD treated cells. Initially, MDA-MB-231 cells (1 × 10^4^ per well) were seeded into a 12-well plate. After 24 h, the cells were treated with 50 ppm Pd of Pd@COS-RGD for 4 h, and then, PTT was performed under 808-nm laser at 2 W cm^−2^ for 5 min. After PTT, the cells were allowed to incubate for 3 h and then stained with AO/PI. Laser-exposed cells and Pd@COS-RGD treated cells without laser exposure were also stained with AO/PI to serve as controls. Finally, the stained cells were observed under a fluorescent microscope (Leica Microsystems, Wetzlar, Germany) and images were captured.

### Flow cytometric analysis of cell death

The pre-seeded cells were incubated with Pd@COS-RGD for 4 h and exposed to an 808-nm laser at 2 W cm^−2^ for 5 min. Treated cells were incubated in a CO_2_ incubator for 8 h at 37 °C. Then, the live and dead cells were harvested and washed with cold PBS, then stained with FITC-Annexin V Apoptosis detection kit (BD Pharmingen^TM^, USA) according to the manufacturer’s protocol. Cell death was analyzed using flow cytometry (BD Biosciences, CA, USA) according to the manufacturer’s directions. The acquired data was analyzed using FlowJo software (Ashland, Oregon, USA).

### Mouse models

All the animal experiments were performed in accordance with protocols approved by Pukyong National University Animal Care and Use Committee. Six-week old female BALA/c nude mice weighing 18–20 g were obtained from Orient Bio Inc. (Gyeonggi-Do, Korea) and used for the investigation. The animals were housed in stainless steel cages under standard conditions (20 ± 2 °C room temperature, 60 ± 10% relative humidity) with a 12 h light/dark cycle. The mice tumor models were prepared by subcutaneous injection of MDA-MB-231 (4 × 10^6^) cells in 100 µL of PBS into the right flank region of the mice. All the *in vivo* tumor xenograft experiments were performed when the tumor size reached ~180 mm2.

### Biodistribution of Pd@COS NPs and Pd@COS-RGD

To analyze the enhanced accumulation of Pd@COS-RGD in tumor region, the nude mice bearing tumor were divided into two groups and given tail vein injections of 200 µL of Pd@COS NPs or Pd@COS-RGD (200 µg) separately. The animals were sacrificed at 1, 12, and 24 h time intervals. Then, the tumor region was dissected and lysed with aqua regia overnight. The Pd content in each group was quantified using inductively coupled plasma mass spectrometry (ICP-MS). To determine the biodistribution in major organs, major organs such as the heart, liver, spleen, lung, and kidney were dissected after 24 h of tail vein injection. Then, the organs were lysed in aqua regia, and Pd was quantified by ICP-MS analysis.

### *In vivo* PTT

The animals were divided into four groups for *in vivo* PTT as follows (n = 5): group I, PBS; group II, PBS + 808-nm laser treatment; group III, Pd@COS-RGD NPs; group IV, Pd@COS-RGD + 808-nm laser treatment. The animals in groups III and IV received 200 µL of (5 mg/kg) Pd@COS-RGD by tail vein injection. On hour after injection, the tumor regions of the animals in groups II and IV were irradiated with an 808-nm laser (2 W cm^−2^) for 5 min. Simultaneously, the temperature level was recorded and thermographic images were captured using a FLIR i5 infrared camera (FLIR Systems Inc., Portland, USA). Following PTT, tumor sizes were measured using calipers every 2 days and tumor volume was calculated using the following equation.

$${\rm{Tumor}}\,{\rm{volume}}=\frac{{\rm{Tumor}}\,{\rm{length}}\times {({\rm{Tumor}}{\rm{width}})}^{2}}{2}$$

After finalizing the experiments, the animals were sacrificed and their major organs, along with tumor tissue, were removed. The tissues were fixed with 10% neutral buffered formalin for 24 h, processed through a gradient of alcohols, and then embedded in paraffin. The embedded tissues were sliced to ~4–5 µm thickness and stained with hematoxylin and eosin (H&E) stain. The stained tissue sections were observed under a microscope and photographs were taken.

### *In vitro* photoacoustic imaging

A human tissue-mimicking phantom was prepared using 8% polyvinyl alcohol and 0.4% silica in distilled water. The *in vitro* PAT system was developed previously and described by Quang *et al*.^48^. MDA-MB-231 cells were treated with Pd@COS-RGD at concentrations of 10 and 50 ppm for 4 h; control cells were maintained without any treatment. Then, the control and treated cells were separately collected and made into cell inclusions with 4% gelatin. The cell inclusions of the control and treatment groups were loaded into the phantom and the surface was covered with 4% gelation to a depth of 1 cm. A non-ionizing PAT system operating at 808 nm was used to capture images of the cell inclusions in the tissue-mimicking phantom.

### *In vivo* photoacoustic imaging

A non-ionizing PAT system integrated with a tunable optical parametric oscillator (OPO) laser (Surelite III + Surelite OPO Plus, Continuum, CA, USA) was used for generation of photoacoustic (PA) signals. The laser light of the system can be tuned from 650 to 1064 nm with a pulse width of 5 ns and a repetition rate of 10 Hz. The laser light was fixed at 808 nm to obtain PA signals from the mouse tumor injected with Pd@COS-RGD (5 mg/Kg). The incident of laser fluence used to generate the PA signals was fixed below 9 mJ/cm2. The laser beam was launched into the multi-mode optical fiber with a plano-convex lens. The output end of the fiber was aligned to a focused transducer (Olympus NDT, Waltham, MA, USA). The PA signals generated by this system were captured with the transducer, then digitized and stored using a data acquisition (DAQ) system. Finally, the digitalized PA signals were converted into images using the Hilbert transformation. PAT images were acquired before and after injection of the mouse with Pd@COS-RGD, using 808-nm laser irradiation.

### Statistical analysis

The data are presented as the mean ± standard deviation. Comparisons among groups were performed using OriginPro 8 software (OriginPro8, Northampton, MA, USA) and the statistical significance of differences between groups was assessed using one-way ANOVA analysis.

## Electronic supplementary material

Supplementary Information
