# Supplementary material for: Multimodal tumor-homing chitosan oligosaccharide-coated biocompatible palladium nanoparticles for photo-based imaging and therapy
Source: Sci Rep. 2018 Jan 11;8:500. doi: 10.1038/s41598-017-18966-8 (PMC5764953; doi:10.1038/s41598-017-18966-8)
Supplement: Supplementary file 1 — Supplementary Information [file 41598_2017_18966_MOESM1_ESM.pdf]

Supplementary data

Multimodal tumor homing chitosan oligosaccharide-coated biocompatible palladium nanoparticles for photo-based imaging and therapy

Subramaniyan Bharathiraja[1], Nhat Quang Bui[2], Panchanathan Manivasagan[1], Madhappan Santha Moorthy[1], Sudip Mondal[1], Hansu Seo[2], Nguyen Thanh Phuoc[2], Thi Tuong Vy Phan[2], Hyehyun Kim[2], Kang Dae Lee[3], Junghwan Oh[1,2*]

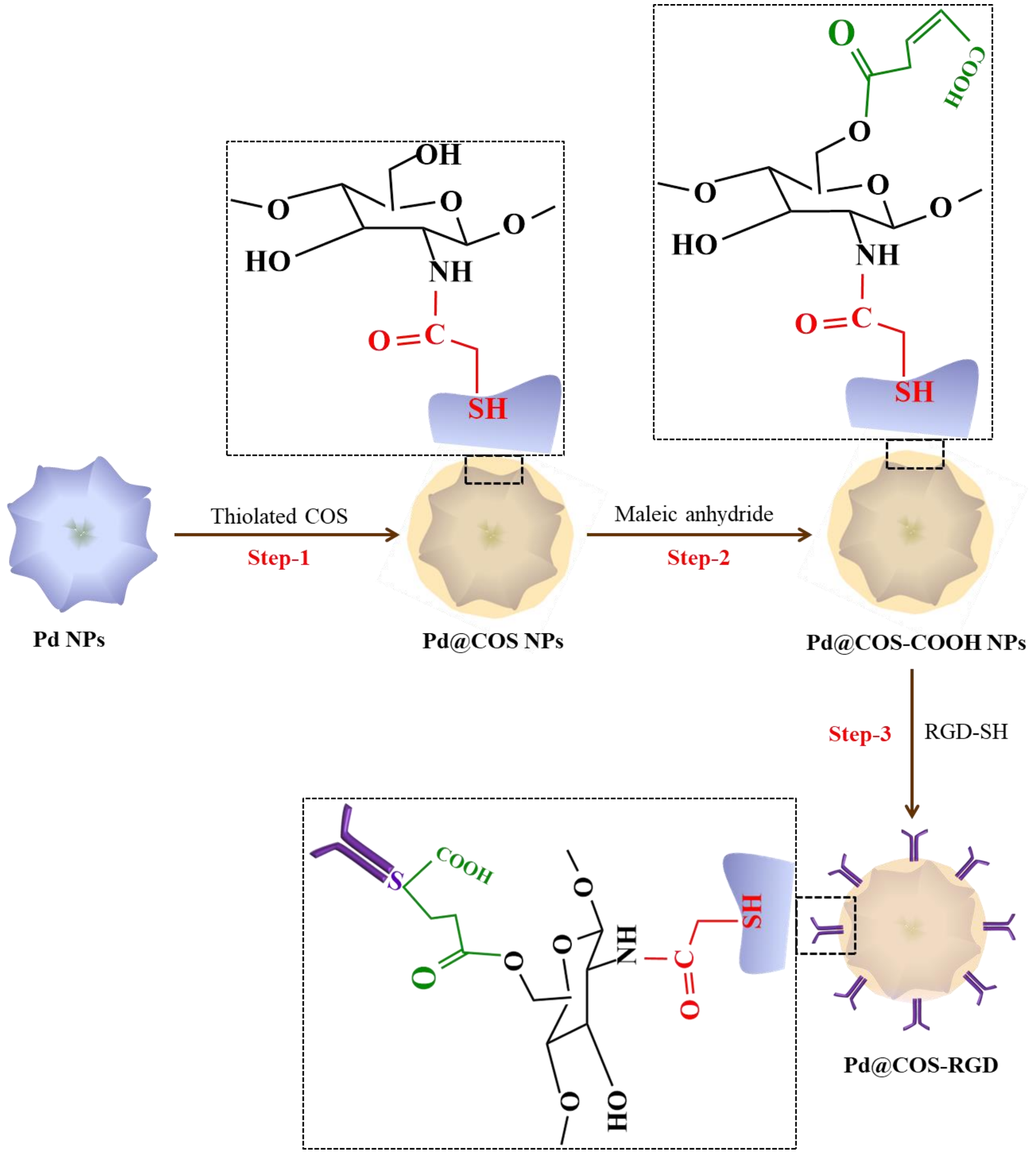

Figure S1. Three step procedure for surface modification of Pd NPs with chitosan oligosaccharide (COS) and further functionalization with RGD peptide to obtain Pd@COS-RGD.

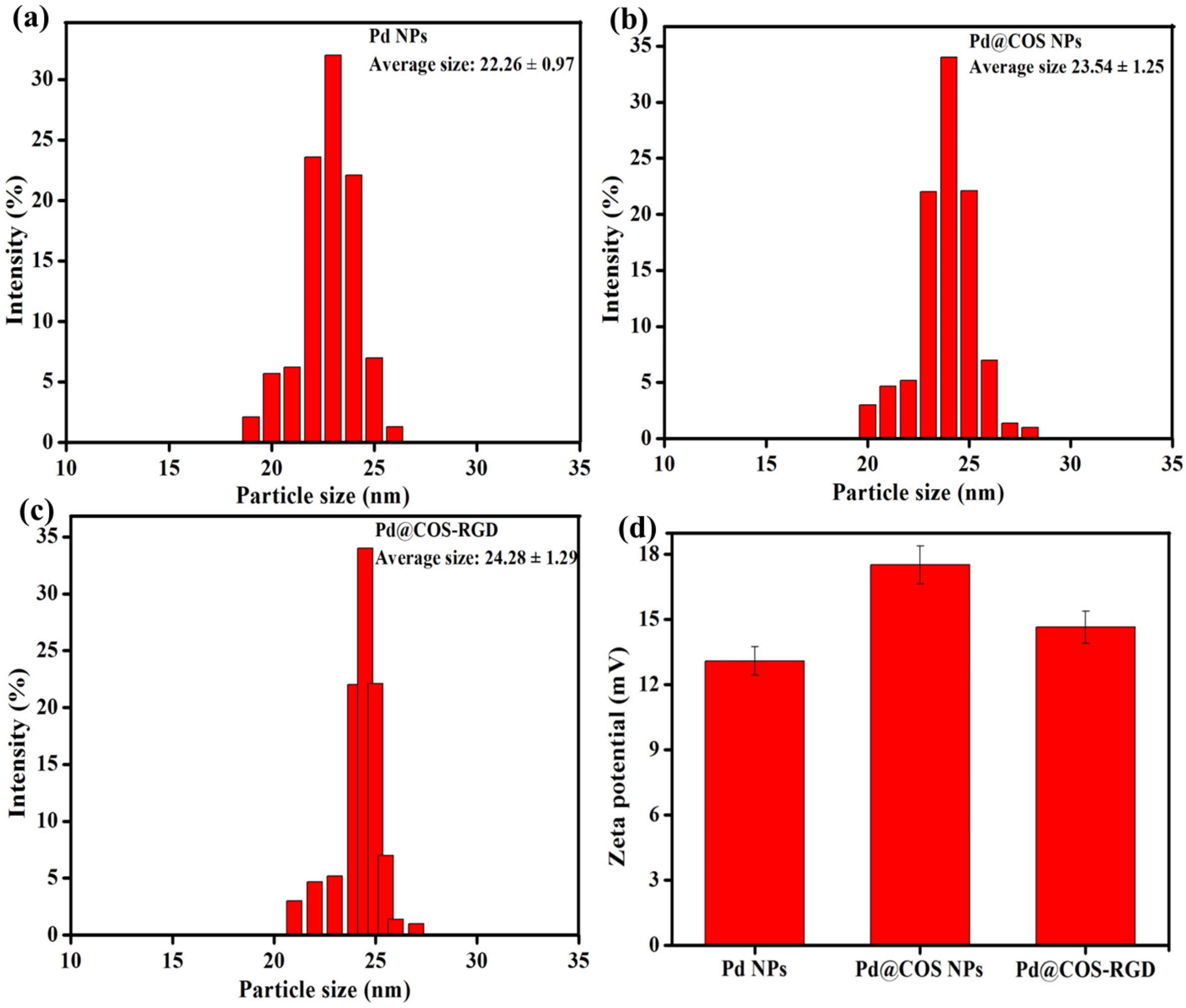

Figure S2. Size distribution of Pd NPs **(a),** Pd@COS NPs **(b)** and Pd@COS-RGD **(c)**, and corresponding zeta potential of each particles **(d).**

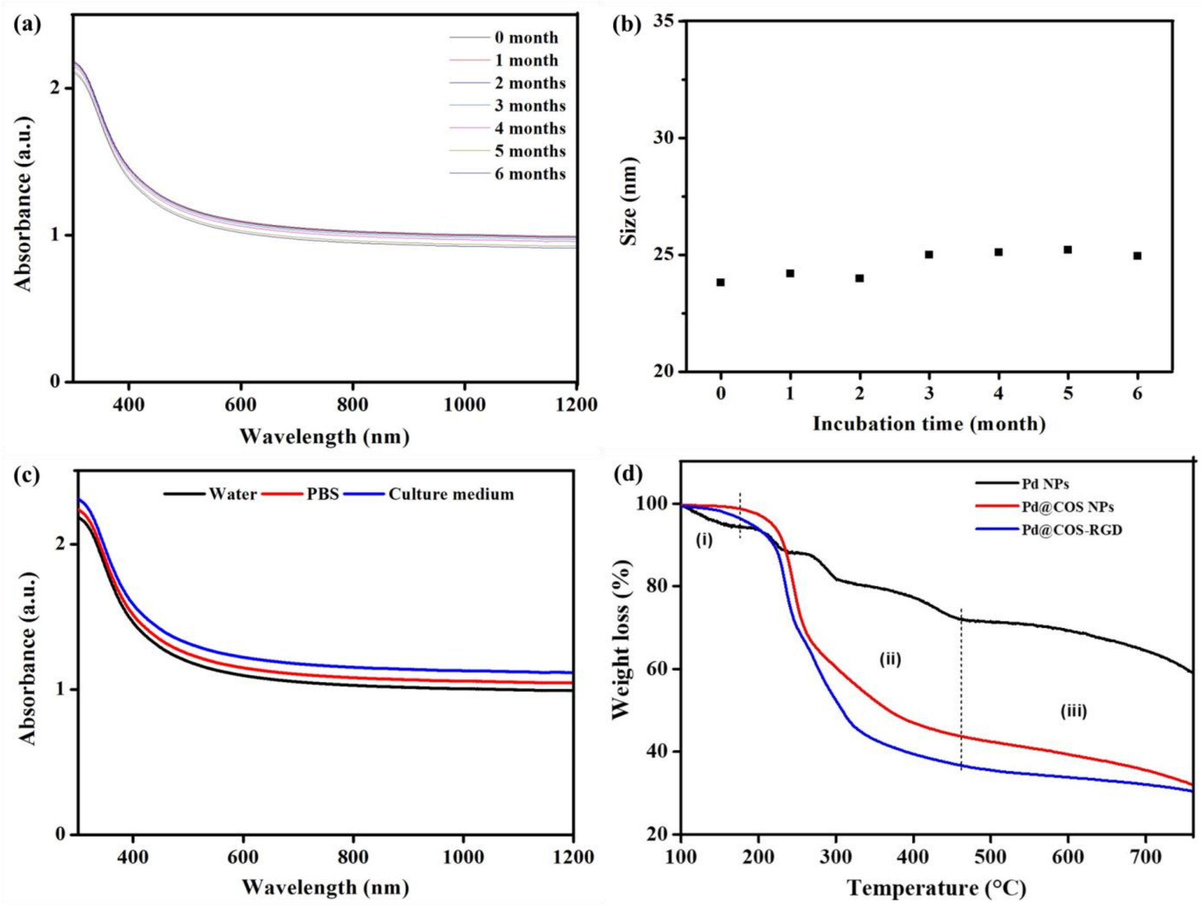

Figure S3. Storage stability of Pd@COS-RGD over 6 months in water analysed by UV-Vis absorbance spectrum **(a)** and size distribution analysis **(b)** over the period of 6 months. **(c)** The stability of Pd@COS-RGD in different medium. **(d)** Thermogravimetric analysis of Pd NPs, Pd@COS NPs and Pd@COS-RGD.

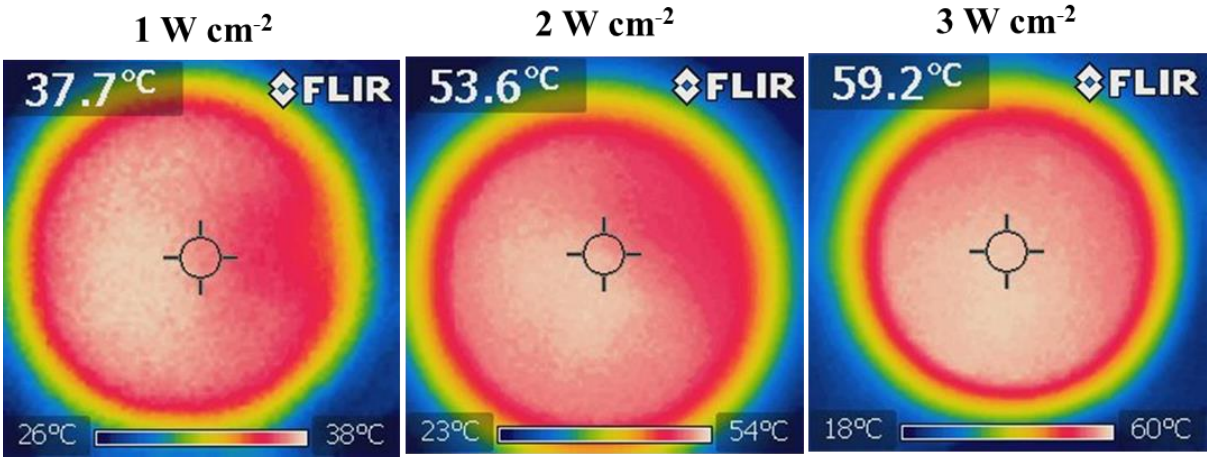

Figure S4. Infrared thermographic images of Pd@COS-RGD (50 ppm) in 12 well plate under 808 nm laser irradiation at different power densities.

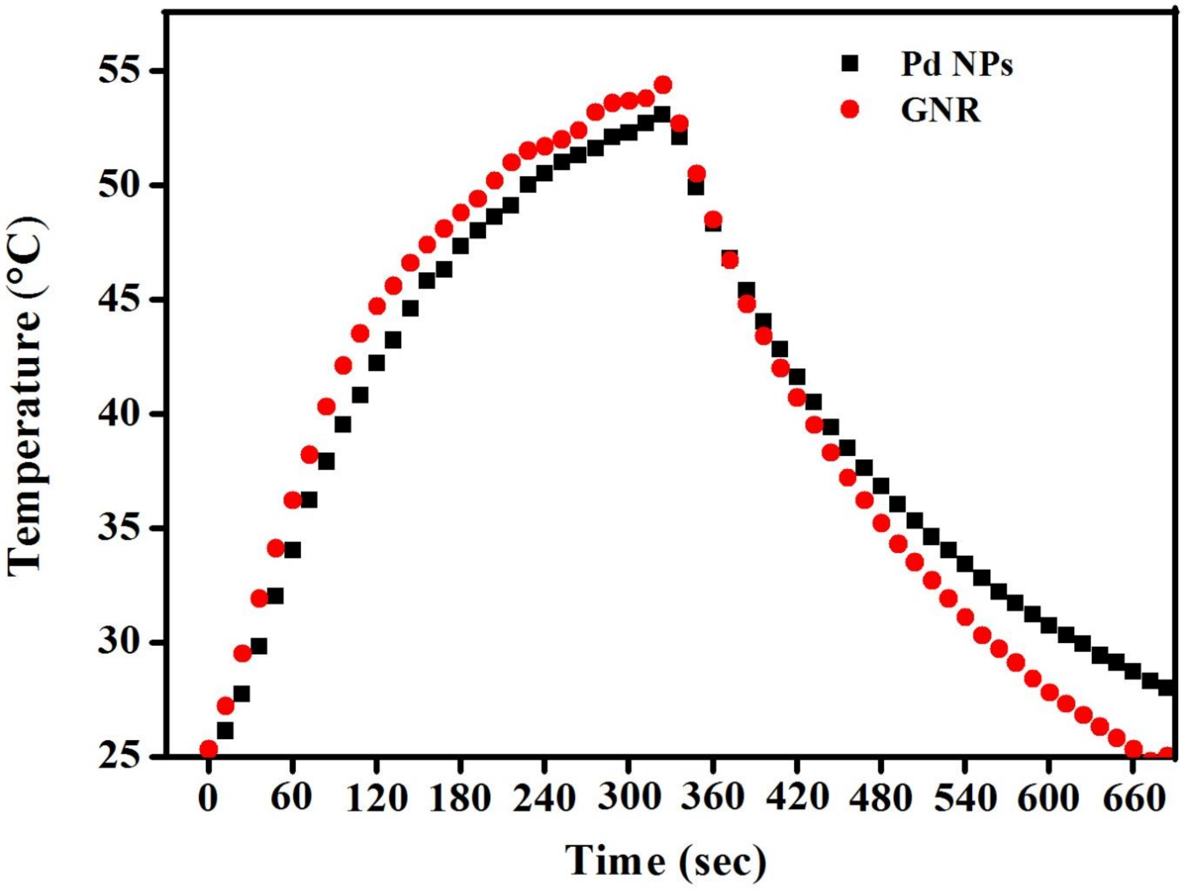

Figure S5. Thermal plots of heating and cooling process for Pd NPs and GNR dispersed in aqueous and irradiated under 808 nm laser at 2 W cm^{-2}.

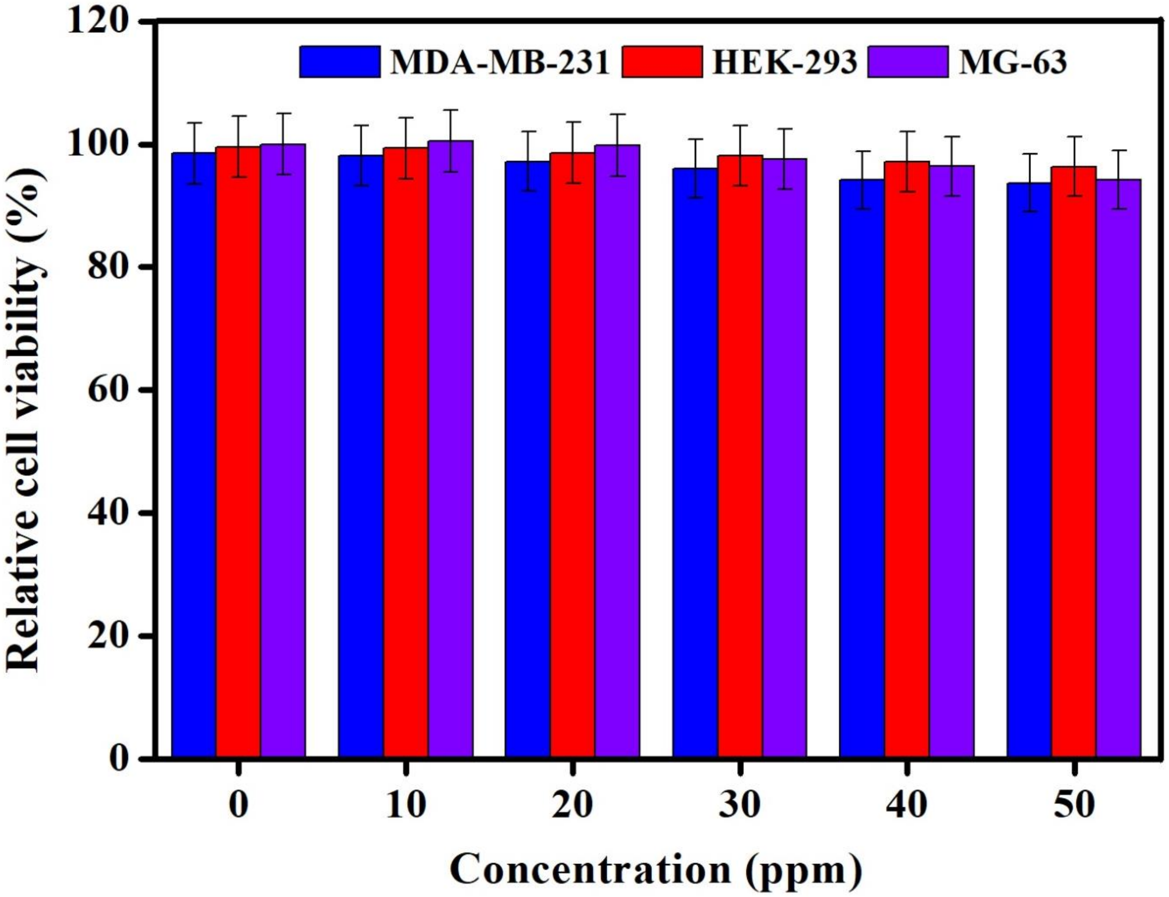

Figure S6: In Vitro biocompatibility of Pd@COS-RGD with different cell lines for 24 h analysed by MTT assay.

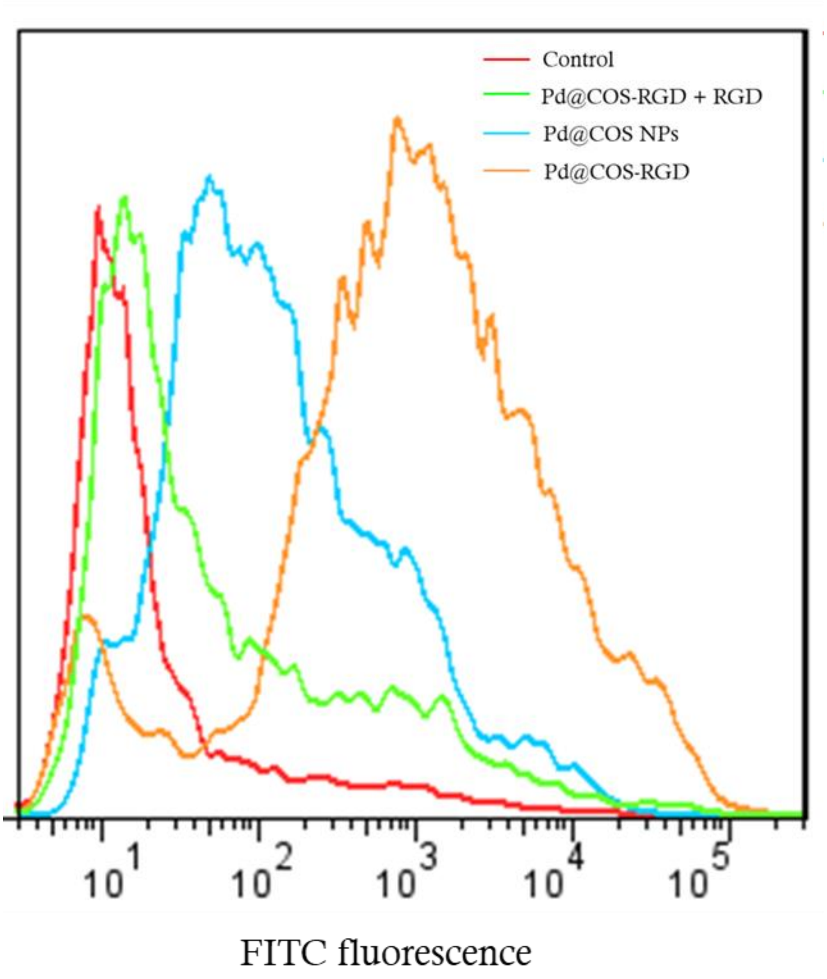

Figure S7: Cellular uptake of Pd@COS NPs and Pd@COS-RGD by MDA-MB-231 cells using FACS analysis. The particles were labelled with FITC and incubated with cells for 1 h.

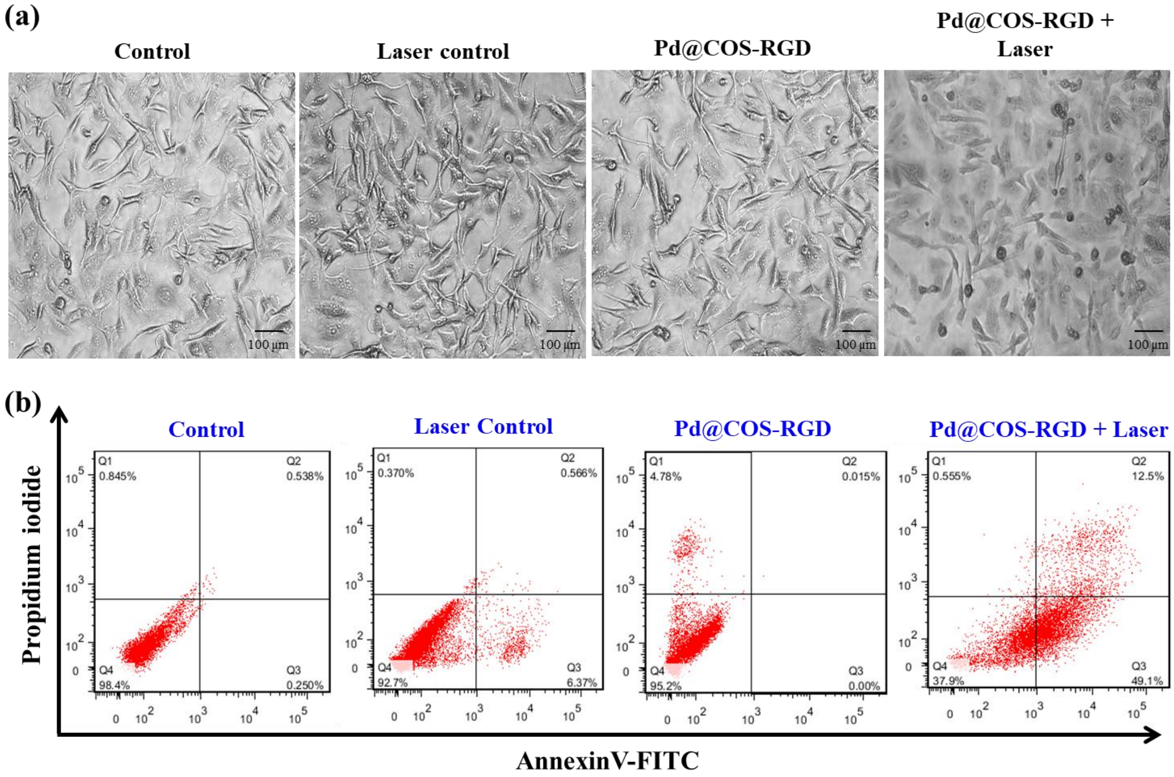

Figure S8. (a) Bright field images of MDA-MB-231 cells in different conditions and PTT therapy performed under 808 nm laser at 2 W cm^{-2} for 5 min. **(b)** FACS analysis of cell death after 8 h of PTT therapy under 808 nm laser at 2 W cm^{-2} power density.

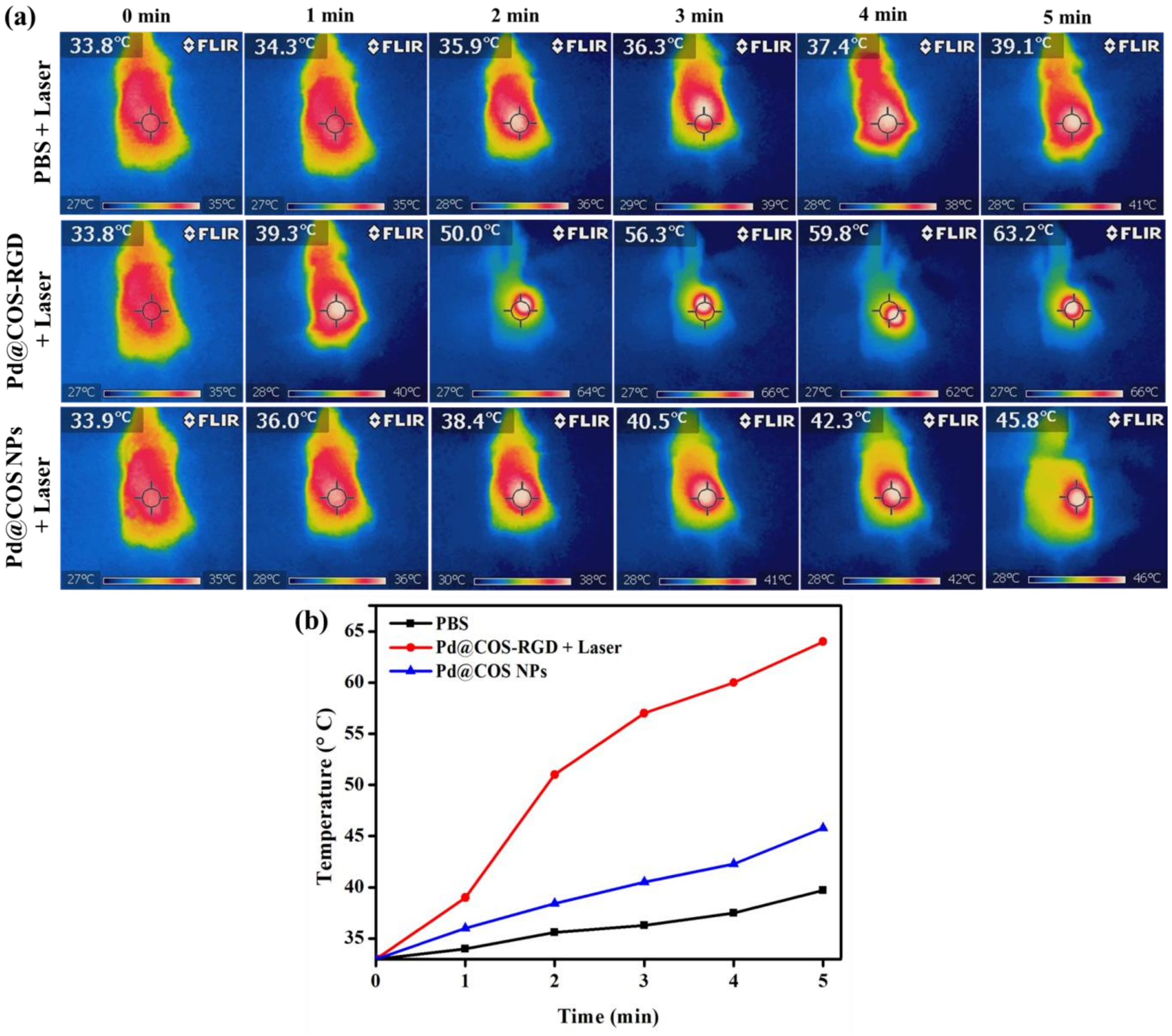

Figure S9. (a) Thermal images of MD-MB-231 tumor bearing mouse exposed to 808 nm laser for 5 min after 1 h of tail vein injection of PBS, Pd@COS NPs and Pd@COS-RGD. **(b)** Temperature curve of in vivo tumor upon 808 nm laser irradiation at 2 W cm^{-2} power density.

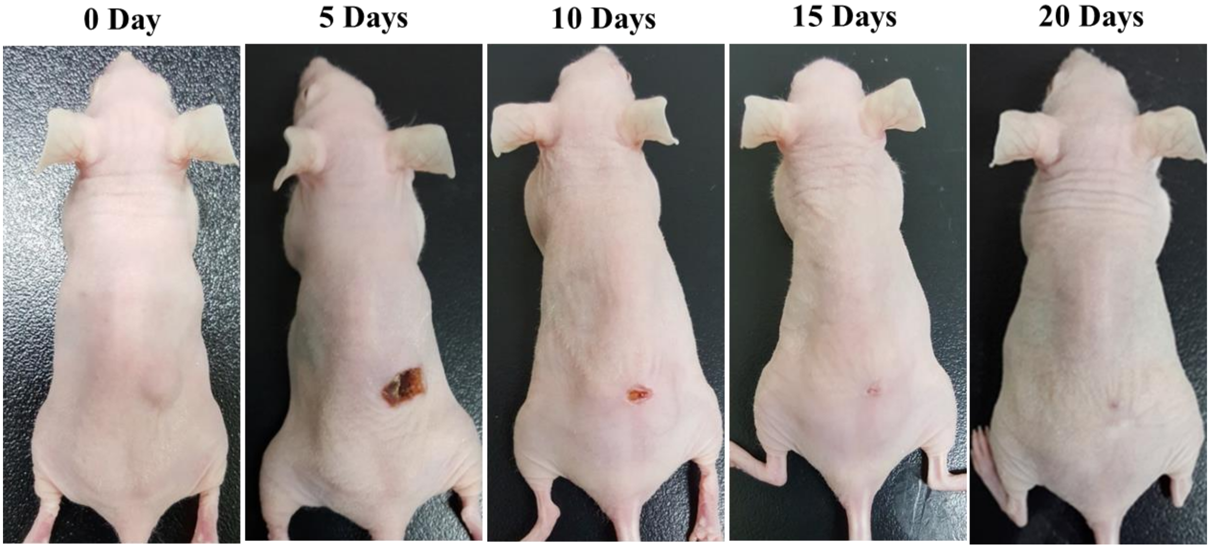

Figure S10. Photograph of MDA-MB-231 tumor curing in nude mice after PTT treatment by injecting Pd@COS-RGD. The 808 nm laser was used for PTT treatment at 2 W cm^{-2} power density.

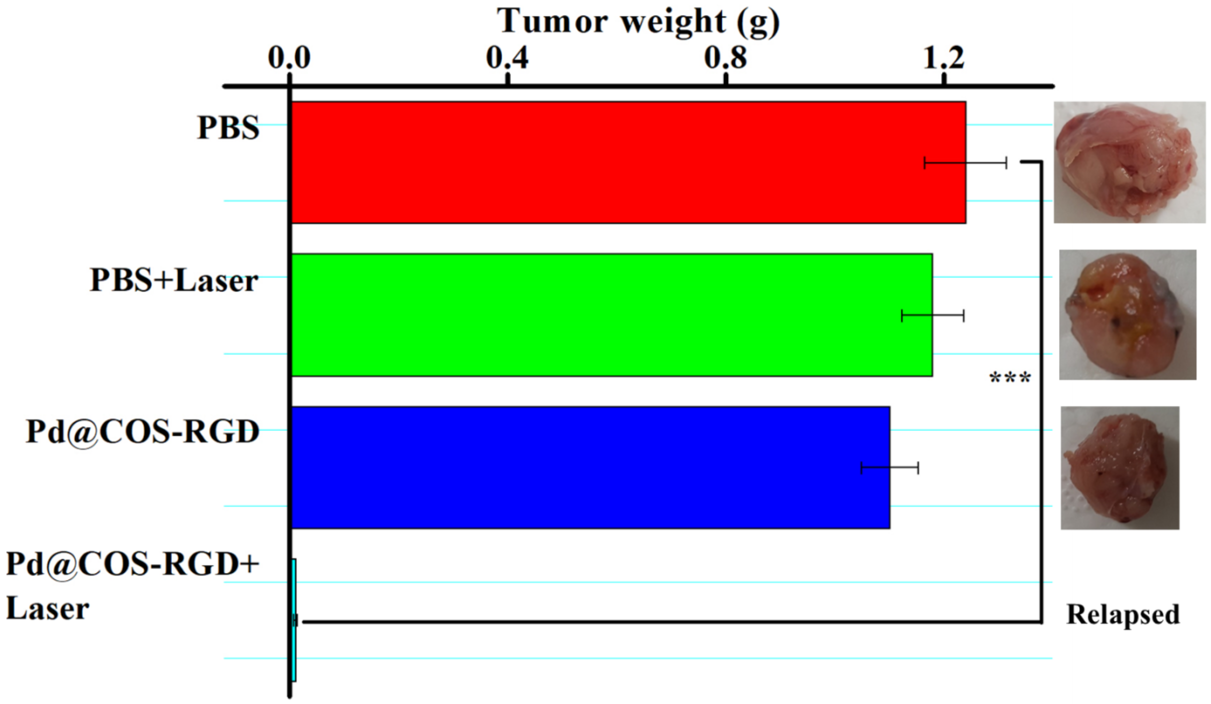

Figure S11. The weight of tumor in different groups BALB/c nude mice after 20 days of PTT treatment (808 nm laser, 2 W cm^{-2}, 5 min) along with tumor images obtained from sacrificed mice at the end of experiment. Results were presented as mean ± standard deviation. (*** $p<0.001$).

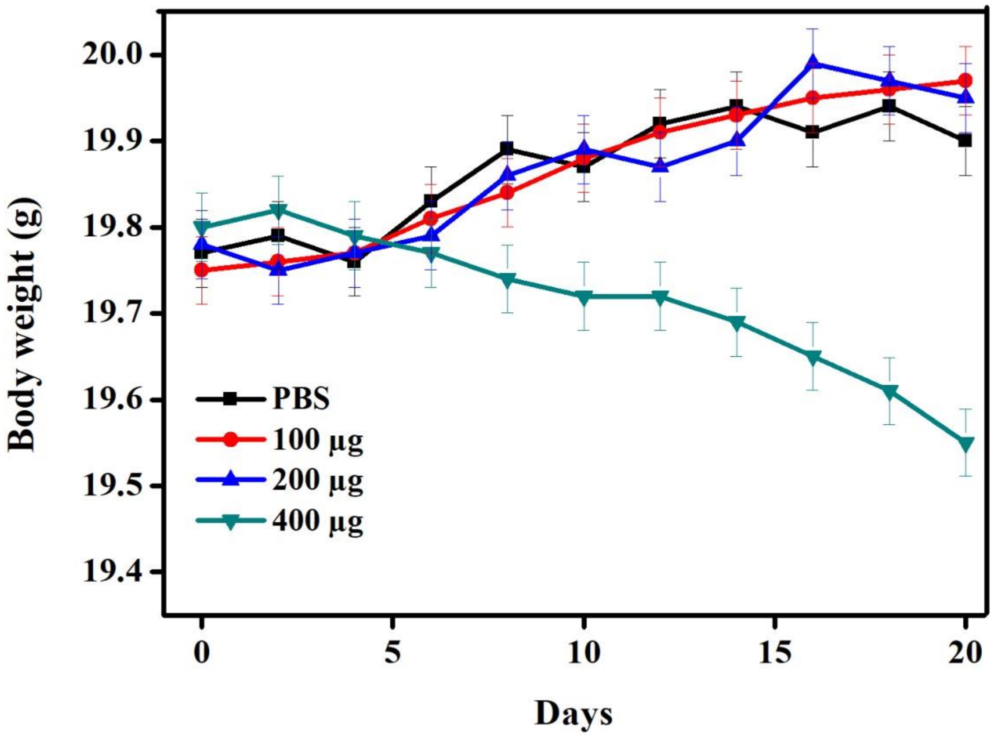

Figure S12: The body weight of mice treated with different concentration of Pd@COS-RGD (Values represent means ± SE).

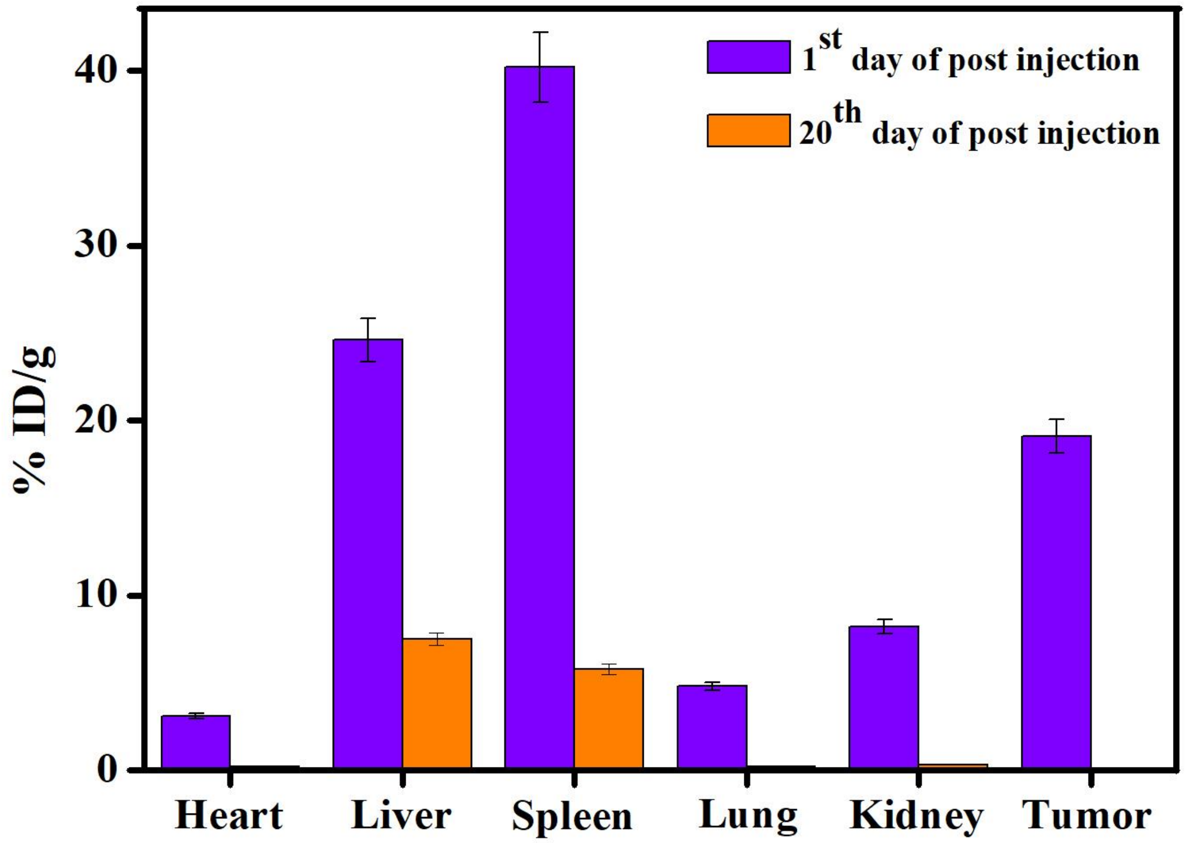

Figure S13. Biodistribution of Pd@COS-RGD in major organs and tumor of BALB/c nude mouse. Results were presented as mean ± standard deviation.

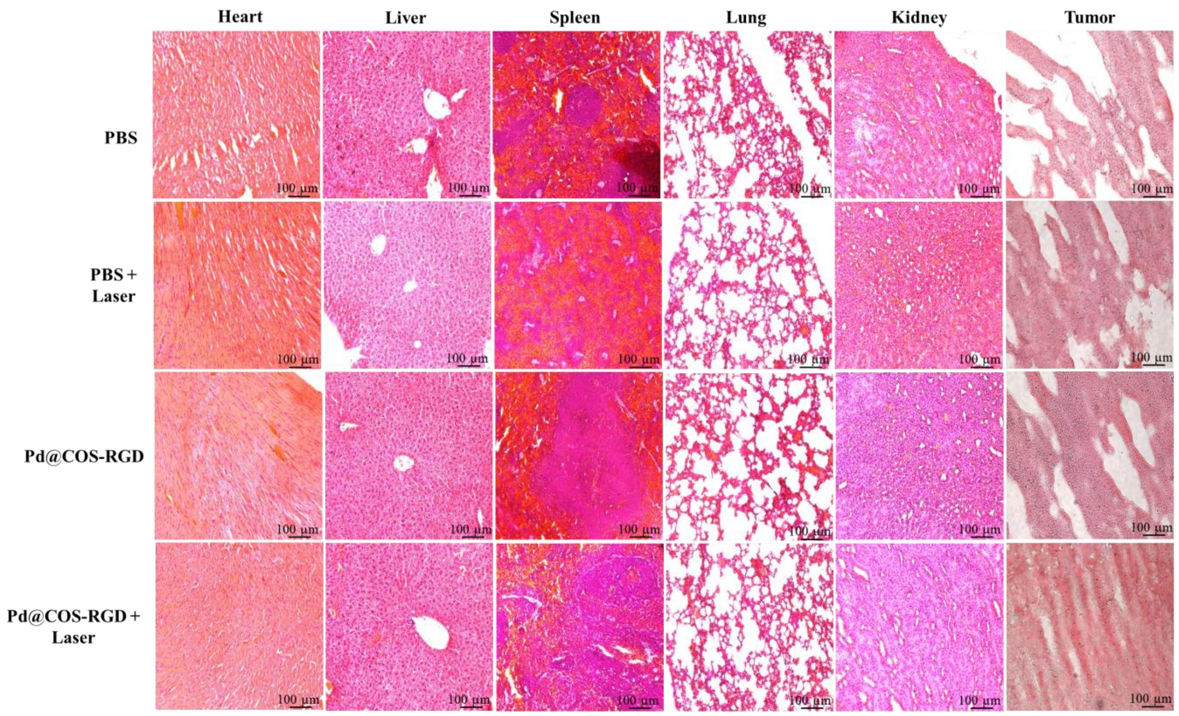

Figure S14. Histology of major mouse organs and tumor from different treatment groups using hematoxylin and eosin stain after 20 days of laser treatment.
